# Exploring Cloned Disease Resistance Gene Homologues and Resistance Gene Analogues in *Brassica nigra*, *Sinapis arvensis*, and *Sinapis alba*: Identification, Characterisation, Distribution, and Evolution

**DOI:** 10.3390/genes16080849

**Published:** 2025-07-22

**Authors:** Aria Dolatabadian, Junrey C. Amas, William J. W. Thomas, Mohammad Sayari, Hawlader Abdullah Al-Mamun, David Edwards, Jacqueline Batley

**Affiliations:** 1 School of Biological Sciences, The University of Western Australia, Perth, WA 6009, Australia; junrey.amas@uwa.edu.au (J.C.A.); william.thomas@uwa.edu.au (W.J.W.T.); dave.edwards@uwa.edu.au (D.E.); jacqueline.batley@uwa.edu.au (J.B.); 2 Department of Plant Science, University of Manitoba, Winnipeg, MB R3T 2N2, Canada; mohammad.sayari@umanitoba.ca; 3 Centre for Applied Bioinformatics, The University of Western Australia, Perth, WA 6009, Australia; 4 The UWA Institute of Agriculture, The University of Western Australia, Perth, WA 6009, Australia

**Keywords:** Brassicaceae, resistance gene analogues, weed, homologues, phylogenetic

## Abstract

This study identifies and classifies resistance gene analogues (RGAs) in the genomes of *Brassica nigra*, *Sinapis arvensis* and *Sinapis alba* using the RGAugury pipeline. RGAs were categorised into four main classes: receptor-like kinases (RLKs), receptor-like proteins (RLPs), nucleotide-binding leucine-rich repeat (NLR) proteins and transmembrane-coiled-coil (TM-CC) genes. A total of 4499 candidate RGAs were detected, with species-specific proportions. RLKs were the most abundant across all genomes, followed by TM-CCs and RLPs. The sub-classification of RLKs and RLPs identified LRR-RLKs, LRR-RLPs, LysM-RLKs, and LysM-RLPs. Atypical NLRs were more frequent than typical ones in all species. Atypical NLRs were more frequent than typical ones in all species. We explored the relationship between chromosome size and RGA count using regression analysis. In *B. nigra* and *S. arvensis*, larger chromosomes generally harboured more RGAs, while *S. alba* displayed the opposite trend. Exceptions were observed in all species, where some larger chromosomes contained fewer RGAs in *B. nigra* and *S. arvensis*, or more RGAs in *S. alba*. The distribution and density of RGAs across chromosomes were examined. RGA distribution was skewed towards chromosomal ends, with patterns differing across RGA types. Sequence hierarchical pairwise similarity analysis revealed distinct gene clusters, suggesting evolutionary relationships. The study also identified homologous genes among RGAs and non-RGAs in each species, providing insights into disease resistance mechanisms. Finally, RLKs and RLPs were co-localised with reported disease resistance loci in *Brassica,* indicating significant associations. Phylogenetic analysis of cloned RGAs and QTL-mapped RLKs and RLPs identified distinct clusters, enhancing our understanding of their evolutionary trajectories. These findings provide a comprehensive view of RGA diversity and genomics in these Brassicaceae species, providing valuable insights for future research in plant disease resistance and crop improvement.

## 1. Introduction

The genus *Brassica*, which belongs to the Brassicaceae family, encompasses a diverse range of species [1]. It has been the focus of extensive breeding efforts to enhance the agronomic traits, yield and resilience to a wide range of biotic and abiotic stresses [2]. Biotic stressors, including fungal, bacterial, and oomycete pathogens, pose a major challenge to global food security and sustainable agriculture [3]. Various pathogens, including *Leptosphaeria* species (*L. maculans*, *L. biglobosa*), *Sclerotinia sclerotiorum*, *Albugo candida*, *Hyaloperonospora* species (*H. parasitica*, *H. arabidopsidis*), *Pseudomonas syringae*, *Plasmodiophora brassicae*, *Xanthomonas* spp., *Fusarium oxysporum* matthioli, *Botrytis cinerea*, *Erysiphe cichoracearum*, and *Alternaria* species (*A. brassicicola*, *A. brassicae*), impose limitations on the production of Brassicaceae species, particularly crop members [4,5,6,7]. These diseases not only limit crop production but also have a significant economic impact and threaten global food security by reducing yield and quality.

Plants, including *Brassica* species, have evolved an intricate defence system to protect themselves from pathogens, including bacteria, fungi, viruses, and pests. Pathogen-associated molecular pattern (PAMP)-triggered immunity (PTI) and effector-triggered immunity (ETI) are two crucial pathways within the immune system that contribute to both qualitative and quantitative resistance against pathogens [8]. Resistance genes play a central role in this sophisticated defence system as critical components of a plant’s immune response. These genes function as sentinels, guarding the plant and are ready to trigger a defence response upon pathogen detection. Resistance gene analogues (RGAs) are involved in plant defence mechanisms as the genetic arsenal against various pathogens. These genes contain conserved domains [9] which have been implicated in pathogen recognition. The most common classes of RGAs in plants are nucleotide-binding site-leucine-rich repeats (NBS-LRRs or NLRs) and transmembrane-leucine-rich repeats (TM-LRRs). Receptor-like kinases (RLKs) and receptor-like proteins (RLPs) are subclasses of TM-LRR proteins that share similar structural features but exhibit functional diversity [9,10]. Each of these classes plays a distinct role in safeguarding the plant. NLRs primarily serve as cytoplasmic receptors and are involved in ETI and trigger plant defence by recognising specific pathogen effectors, such as *RPS2*, which detects the *AvrRpt2* effector from *P*. *syringae* [11]. RLKs and RLPs participate in PTI, which relies on pattern recognition receptors (PRRs) [12] and act as the first line of defence by recognising pathogen elicitors, for example, the *FLS2* receptor detects bacterial flagellin [13]. They also contribute to various aspects of plant development [14,15]. NLRs comprise subtypes like coiled-coil (CC)-NBS (CN), CNL, NBS, NBS-LRR (NL), Toll/Interleukin-1 receptor (TIR)-NBS-LRR (TNL), TIR-NBS (TN), TIR with unknown domains (TX), and NLRs with other domains (Other-NLR) [8,16,17]. In contrast, RLKs, which include subcategories such as LRR-RLK, Lysin motif (LsyM) (LysM-RLK), and other receptor (Other-RLK), along with RLPs, which encompass subclasses like LRR-RLP and LysM (LysM-RLP), serve not only as the initial line of defence by recognising pathogen elicitors [12,14] but also contribute to plant development [15,18]. The implications for crop protection are significant, underscoring the need for a more in-depth examination of the interaction between these gene families. Understanding the functions of these resistance (*R*) genes is crucial for developing effective strategies to enhance crop disease resistance.

Given the central role of these *R* genes in plant immunity, there is growing interest in identifying novel resistance sources from less-studied, wild Brassicaceae species. Wild species within the Brassicaceae family offer valuable genetic resources for crop improvement, particularly in identifying novel resistance *R* genes against pests and pathogens [19]. These wild relatives often exhibit traits not found in cultivated varieties, having naturally evolved mechanisms to combat local pathogens and environmental stresses.

*B. nigra*, commonly known as black mustard, serves as a valuable genetic resource due to its resistance to specific pests and pathogens. Notably, it displays high resistance against all local *P. brassicae* pathotypes in Canada [20,21,22]. The Brassica B genome is known to harbour *R* genes against many pathogens. For instance, novel sources of blackleg resistance genes have been identified within the B genome of *B. nigra* [23], which, as a progenitor species of *B. juncea* and *B. carinata*, more easily facilitates the transfer of *R* genes into these cultivated species, providing valuable sources of resistance. Similarly, closely related members of the Brassicaceae family, such as *Sinapis alba* (white mustard) and *S. arvensis* (wild mustard), also harbour significant genetic diversity with potential for crop improvement. These wild mustard species, which share a common ancestry with cultivated *Brassica* species, coexist in agricultural ecosystems and have likely evolved mechanisms to combat local pathogens, including fungal diseases such as *L. maculans*. Breeders can introduce genetic diversity into cultivated *Brassica* varieties by identifying *R* genes in *S. alba* and *S. arvensis*, enhancing their resilience to diseases. The genetic resources from *B. nigra* and these wild mustard species provide a powerful tool for reducing reliance on chemical fungicides, thereby supporting sustainable agricultural practices.

With next-generation sequencing and computational analyses, the identification and characterisation of RGAs in plant genomes has become increasingly accessible. Genome-wide scans have successfully detected *R* genes in various plant species, revealing the presence of thousands of RGAs, with variable numbers in different species [10].

Despite the recognised potential of wild Brassicaceae species as sources of novel resistance genes, a comprehensive genome-wide characterisation of *R* genes and their relationship to known disease resistance loci remains largely unexplored. By exploring the genetic diversity of wild species, breeders can introduce these *R* genes into cultivated *Brassica* crops, thereby enhancing their resilience to biotic stresses and reducing their dependence on chemical controls. This approach supports sustainable agriculture by improving yield stability and enhancing resilience in *Brassica* production systems.

In this study, unlike prior work that has focused mainly on cultivated species, we applied RGAugury to the genomes of *B. nigra*, *S. arvensis* and *S. alba* to systematically identify RGAs within their genomes. We aimed to determine the types, chromosomal distribution and patterns of physical clustering of RGAs. By characterising these genes, we can uncover potential candidates for further functional analysis and utilisation in breeding programmes to enhance disease resistance in *Brassica* crops. We also utilised the protein sequences of 49 cloned *R* genes that have been verified for their effectiveness against fungal and bacterial diseases [24]. We aimed to identify the homologues of these genes, known as cloned disease resistance gene homologues (CDRHs), in *B. nigra*, *S. arvensis* and *S. alba*. Finally, we examined the phylogenetic relationship of RLK RGAs and analysed their evolutionary relationship with CDRHs. Our research findings have the potential to expedite the discovery and cloning of functional RGAs, ultimately enhancing disease resistance in *Brassica* through breeding programmes.

## 2. Materials and Methods

### 2.1. Reference Genomes

The *B. nigra*, *S. arvensis* and *S. alba* genomes used in this study were downloaded from figshare [25]. The genome of *B. nigra*, with a GC content of 38.3%, comprises eight chromosomes (2n = 16) and 409 contigs, housing a total of 54,886 genes (~515 Mb). *S. arvensis* features a genome with a GC content of 37.4%, distributed across nine chromosomes (2n = 18) and 780 contigs, encompassing 48,466 genes (~450 Mb). The *S. alba* genome, with a GC content of 37.1%, spans 12 chromosomes (2n = 24) and 468 contigs and contains 41,132 genes (~436 Mb).

### 2.2. Genome-Wide Mining of Resistance Gene Analogues

The RGAugury pipeline (Version 2.2 Docker Image), a computational tool for the comprehensive prediction of RGAs throughout the genome [26] was used following the instructions provided in the official repository (https://bitbucket.org/yaanlpc/rgaugury/wiki/Home, accessed on 15 June 2025) to perform in silico prediction of RGAs and their subfamilies in the genomes of *B. nigra*, *S. arvensis* and *S. alba*. For domain prediction, RGAugury was run with InterProScan version 5, which integrates multiple domain and motif databases, including Pfam version 36.0. Hidden Markov model searches within InterProScan were performed using HMMER version 3.3.2 to ensure accurate domain assignments. The identified RGAs were categorised into four primary classes: RLK (receptor-like kinases), RLP (receptor-like proteins), NLR (nucleotide-binding domain and leucine-rich repeat) and TM-CC (transmembrane-coiled-coil) genes. The NLR gene family members were further subdivided into groups based on their specific domain architecture. These subgroups included CNL (CC-NBS-LRR), TNL (TIR-NBS-LRR), TN (TIR-NBS), CN (CC-NBS), NL (NBS-LRR), TX (TIR with unclassified domains) and OTHER. In cases where genes possessed RPW8 domains, their classifications were manually reassigned; genes initially categorised as NBS with an RPW8 domain were reclassified as RN, those initially classified as NL with an additional RPW8 domain were reclassified as RNL, and all other genes (TNL, CNL, CN, TN, and TX) with an additional RPW8 domain were reclassified as OTHER [10]. Within the NLR subgroup, TNL, CNL, and RNL were collectively referred to as typical NLRs, while the remaining subgroups containing partial or disordered domains were labelled as atypical NLRs. To further refine the classification, RLKs and RLPs were divided into three distinct subclasses: PRRs containing an LRR domain (LRR-RLK/RLP), PRRs containing Lys motifs (LysM-RLK/RLP) and PRRs with any other domain (other-RLK/RLP) [10].

### 2.3. Characterisation of Resistance Gene Analogues

The number of different RGAs and the relationship between the number, chromosome and genome size were calculated using a custom Python script using seaborn and matplotlib libraries (https://github.com/Aria-Dolatabadian/Relationship-between-chromosome-and-genome-size-with-RGA-numbers/tree/main, accessed on 15 June 2025). The ChromoMap v0.2 R package [27] was used to calculate and visualise the RGA candidates’ density and distribution in *B. nigra*, *S. arvensis* and *S. alba*. The circos plots were generated using Circa (http://omgenomics.com/circa, accessed on 15 June 2025). A Python script (https://github.com/Aria-Dolatabadian/Genes-Physical-Clustering-, accessed on 15 June 2025) was developed to execute the physical clustering of RGAs. This clustering process was facilitated using various libraries, including Pandas, Matplotlib, and NumPy. The criteria for defining clusters involved grouping RGAs within a distance threshold of 10 kilobases (kb). The physical clustering was performed either on all the RGAs collectively or as separate analyses for each family. A custom Python script (https://github.com/Aria-Dolatabadian/Genes-Hierarchical-Clustering-Dendrogram/blob/main/Code2.py, accessed on 15 June 2025) was employed to carry out a comprehensive assessment of pairwise similarities among sequences of RGAs. To visually represent these similarities, dendrograms were created using the itertools, NumPy, Matplotlib, and SciPy libraries.

### 2.4. Mining the Protein Sequences of the Cloned Genes

Prior studies [24,28] have compiled a list of 49 cloned *R* genes found in *Brassica* crop species and *Arabidopsis* (42 from *Arabidopsis* and seven from *Brassica* species), known to provide resistance against diseases, such as *Alternaria* black spot (ABS), blackleg (BL), black rot (BR), bacterial leaf spot (BLS), clubroot (CR), downy mildew (DM), Fusarium wilt (FW), grey mould (GM), powdery mildew (PM), Sclerotinia stem rot (SSR) and white rust (WR) (Supplementary Table S1). These cloned *R* genes were selected based on the following three criteria: (1) a known gene-for-gene interaction with a corresponding pathogen *Avr* gene, (2) conferring resistance in the form of a hypersensitive response, indicating its involvement in a gene-for-gene interaction, or (3) serving as a helper or accessory gene necessary for the gene-for-gene interaction. These genes offer resistance against fungal and bacterial diseases affecting Brassicaceae species. The protein sequences for these 49 genes were obtained from the UniProtKb and NCBI websites and verified on 26 October 2023.

### 2.5. Homologue Identification

For the homology search, the protein sequences of the 49 cloned genes were aligned against the three genomes using the Protein Basic Local Alignment Search Tool (BLASTp) within Geneious Prime^®^. To ensure the quality of the matches, the criteria previously employed in studies that identify homologous genes in plants were applied [28,29]. Specifically, BLASTp hits with E values falling outside the range of E0 to E-45 or those with less than 60% similarity were excluded from further analysis. Additionally, since the smallest reference gene in this study, *At_Rpw8.1*, comprised 148 amino acids (aa), any BLASTp hits with fewer than 148 aa were also excluded from subsequent analyses.

### 2.6. Co-Localisation of RLKs and RLPs to Reported Disease Resistance Loci in Brassica Crops

Information about previously identified quantitative trait loci (QTL) associated with disease resistance in *Brassica* crops was collected. This included 57 QTL, mapped on 21 chromosomes and associated with resistance against blackleg (BL), black rot (BR), clubroot (CR), hypocotyl rot (HR), *Sclerotinia* stem rot (SSR), and white rust (WR) (Table 1). These QTL and the regions in which they overlap are illustrated in Supplementary Figure S1. These QTL and their corresponding genomic positions in *B. juncea* ‘Tumida’ T84-66 v. 1.5 [30], *B. nigra* ‘DH YZ12151’ [30], *B. napus* ‘Darmor bzh’ v. 4.1 [31], and *B. oleracea* ‘TO100’ v. 2.1 [32] were gathered. Subsequently, the presence and quantification of RLKs and RLPs within these genomic regions were determined. The start and end positions of each RLK and RLP were compared against the QTL coordinates using an Excel IF formula to identify whether each gene fell within or outside the QTL regions. Genes located within the QTL boundaries were labelled as “within”, and those outside as “outside”.

### 2.7. Phylogenetic Analysis

Multiple sequences of RLKs and RLPs (found within the QTLs associated with disease resistance) and 49 cloned RGAs were aligned using CLC Genomics Workbench Version 23.0.5. The alignment file was used to build the phylogenetic tree using the UPGMA build method and the Jukes-Cantor protein distance model with 1000 bootstraps. The tree was exported as newick. The online iTOL software [43] (http://itol.embl.de/) was used to read Newick files, generate circular cladograms, and annotate and visualise the phylogenetic trees.

## 3. Results

### 3.1. RGAs Identification and Classification

We employed the RGAugury pipeline to classify RGAs into four main categories within the *B. nigra*, *S. arvensis* and *S. alba* genomes: RLKs, RLPs, NLRs and TM-CC. A total of 4499 candidate RGAs were identified in all three genomes (1625 in *B. nigra* (2.96% of the total genes), 1625 in *S. arvensis* (3.35% of the total genes) and 1249 in *S. alba* (3.03% of the total genes)). The figures in Table 2 and Supplementary Figure S2 display the count and percentage of RGAs on each chromosome and contig, respectively, in the genomes of the three species. In all three genomes, the most prevalent class was RLKs, comprising 821 genes in *B. nigra* (50.52%), 818 genes in *S. arvensis* (50.33%) and 734 in *S. alba* (58.76%), followed by TM-CCs with 272 genes in *B. nigra* (16.73%), 266 genes in *S. arvensis* (16.36%) and 247 genes in *S. alba* (19.77%); and RLPs with 164 genes in *B. nigra* (10.09%), 155 genes in *S. arvensis* (9.53%) and 104 genes in *S. alba* (8.32%) (Table 2).

We further subdivided RLKs and RLPs into three classes based on their N-terminal domains: LRR, LysM, and other receptors. There were 325 and 162 candidate LRR-RLKs and LRR-RLPs in *B. nigra*, 300 and 153 in *S. arvensis*, and 287 and 101 in *S. alba*, respectively. There were 6 LysM- and 2 LysM-RLPs identified in *B. nigra*, 5 and 2 in *S. arvensis* and 6 and 3 in *S. alba*, respectively. There were 490, 513 and 441 Other receptors among RLKs in *B. nigra*, *S. arvensis* and *S. alba*, respectively (Table 2).

Regarding NLR genes, we identified 357 candidates in *B. nigra* (21.96%), 374 in *S. arvensis* (23.01%) and 156 in *S. alba* (12.48%). In *B. nigra*, there were 165 typical NLRs with all three domains (TNL, CNL and RNL) and 192 atypical NLRs with partial or disordered domains (CN, RN, NL, TN, TX and OTHER). These numbers are slightly higher in *S. arvensis*, with 181 typical and 193 atypical NLRs. In *S. alba*, 66 were typical while 90 were atypical NLRs.

The cumulative counts for the three typical NLR classes in *B. nigra* were as follows: TNLs (119), CNLs (37), and RNLs (9). The atypical NLRs were classified into distinct categories: TX (TIR with unclassified domains; 71 genes), TN (TIR-NBS; 30), NL (NBS-LRR; 37), RN (RPW8-NBS; 9), CN (15), and OTHER (30). The combined counts for the three typical NLR classes in *S. arvensis* were as follows: TNLs (132), CNLs (41), and RNLs (8). Similar categories were observed in the atypical NLRs with TX (69), TN (39), NL (33), RN (8), CN (22), and OTHER (22). In *S. alba,* the total counts for the three typical NLR classes were as follows: TNLs (47), CNLs (15), and RNLs (4). For the atypical NLRs, we noted the following categories: TX (37), TN (13), NL (14), RN (7), CN (11), and OTHER (8). Interestingly, in all three species, atypical NLRs outnumbered typical NLRs. There were 11, 12 and RPW8 candidates in *B. nigra*, *S. arvensis* and *S. alba*, respectively (Table 2).

### 3.2. RGA Number and Chromosome Size Relationship

We analysed the relationship between the number of RGAs and chromosome/genome sizes across *B. nigra*, *S. arvensis*, and *S. alba* using regression analysis. As illustrated in Table 2 and Supplementary Figure S3, a general positive correlation was observed in *B. nigra* and *S. arvensis*, where larger chromosomes tended to carry more RGAs. However, this trend was not consistent across all chromosomes. For instance, in *B. nigra*, chromosome 08 was larger than 05 but contained fewer RGAs, representing an exception to the general pattern (Supplementary Table S2). Overall, in *B. nigra*, 11 pairwise comparisons showed that the larger chromosomes contained fewer RGAs. A similar trend was observed in *S. arvensis*, with several chromosomes exhibiting a correlation between size and RGA count, although exceptions existed. For example, chromosome 07 was larger than chromosome 04 but had substantially fewer RGAs (Supplementary Table S2). In *S. arvensis*, 10 pairwise comparisons showed that the larger chromosomes had fewer RGAs. In contrast, *S. alba* did not exhibit a consistent relationship between chromosome size and RGA count. In many instances (30 pairwise comparisons), larger chromosomes had fewer RGAs, suggesting a weaker correlation. For example, chromosome 01, the largest in the genome, contained the fewest RGAs. However, in some cases, larger chromosomes exhibit more RGAs, for instance, chromosomes 09 compared with chromosomes 06. Supplementary Table S2 summarises pairwise comparisons of chromosome sizes and RGA counts across *B. nigra*, *S. arvensis*, and *S. alba*. It shows cases where larger chromosomes contain fewer RGAs, illustrating exceptions to the general correlation trend observed in the regression analysis and Supplementary Figure S3.

### 3.3. RGAs Distribution and Density

**Figure 1:**
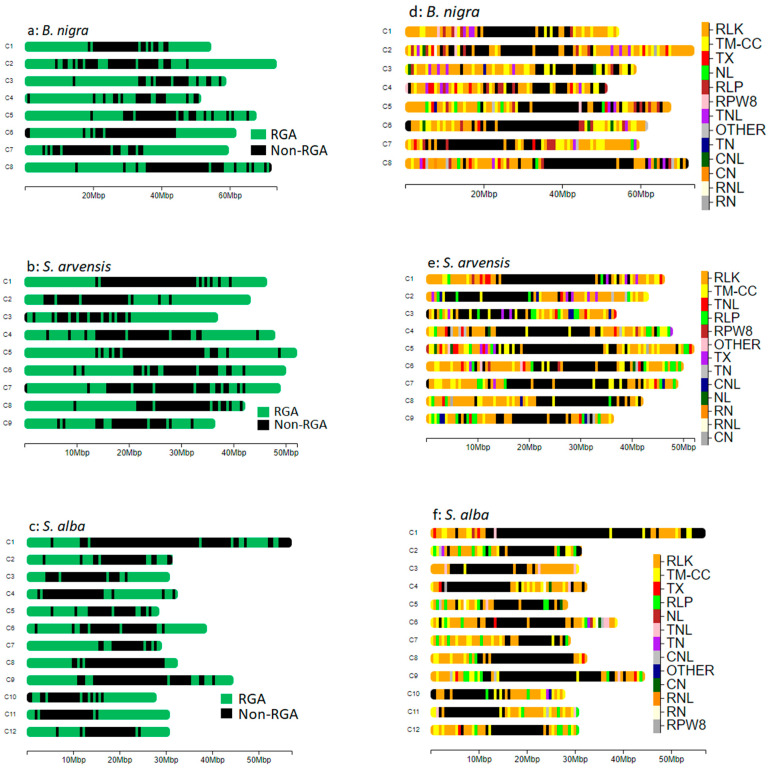
(**a**–**c**): Chromosomal distribution: RGAs are primarily located towards the ends of each chromosome. (**d**–**f**)**:** Differential distribution: Different types of RGAs show varying distribution patterns along the chromosomes, with a notable absence near the centromeres.

We examined the distribution and density of RGAs in the genomes of *B. nigra*, *S. arvensis* and *S. alba*. Generally, RGAs were observed towards the ends of each chromosome (Figure 1a–c). However, different types of RGAs were unevenly distributed along the chromosomes (Figure 1d–f). The distribution and size of RLK, RLP, TM-CC, RPW8 and NLR genes across each chromosome are shown as circos plots in Supplementary Figure S4a–f. The average number of RGAs per chromosome was 201.62 for *B. nigra*, 180.22 for *S. arvensis* and 103.83 for *S. alba*. The relationship between RGA counts and genome size did not show a clear trend across the species, as both *B. nigra* (~515 MB genome) and *S. arvensis* (~450 MB genome) have similar RGA counts (1625), while *S. alba* has a slightly smaller genome (~436 MB) but a noticeably lower RGA count (1249) (Supplementary Figure S3).

In *B. nigra*, chromosomes B02 and B07 contained the most (293, 18.03%) and least (128, 7.87%) number of RGAs, respectively (Table 2). Chromosome B02 had the greatest number of RLKs, with 147 members, while chromosome B07 had the least, with 61 RLKs. Chromosome B05 had the greatest number of RLPs, with 29 members, while chromosome B01 had the fewest RLPs (10). Chromosomes B04 and B07 contained the highest and lowest NLR counts at 91 and 18, respectively. Chromosomes B02 and B01 had the most (53) and least (24) TM-CCs, respectively. The greatest number of RPW8 (4) was found on chromosome B02 (Table 2).

In *S. arvensis*, chromosomes 05 and 01 contained the most (236, 14.52%) and least (129, 7.93%) number of RGAs, respectively (Table 2). Chromosome 06 had the greatest number of RLKs, with 125 members, while chromosome 03 had the least, with 65 RLKs. Similarly, chromosome 06 had the greatest number of RLPs with 27 members, while chromosome 08 had the least RLP number (11). Chromosomes 05 and 01 contained the highest and lowest NLR counts at 120 and 20, respectively. Chromosomes 02 and 01 had the most (38) and least (20) TM-CCs, respectively. The greatest number of RPW8 (4) was found on chromosome 04 (Table 2).

In *S. alba*, chromosomes 04 and 01 contained the most (128, 10.24%) and least (86, 6.88%) number of RGAs, respectively (Table 2). Chromosome 09 had the greatest number of RLKs, with 79 members, while chromosome 08 had the least, with 48 RLKs. Chromosome 12 had the greatest number of RLPs with 15 members, while chromosome 03 did not have any RLP. Chromosomes 04 and 12 contained the highest and lowest NLR count at 28 and 6, respectively. Chromosomes 02 and 05 had the most (35) and least (12) TM-CCs, respectively. The greatest number of RPW8 (3) was found on chromosome 04 (Table 2).

### 3.4. Physical Clustering

In this study, we performed physical clustering analyses for the identified RGAs. The analysis was based on a criterion of genes being considered clustered if they were located within a 10 kb distance from each other. We observed varying degrees of clustering among the genomes and RGAs. In *B. nigra*, when all the RGAs were included in the analyses, 245 RGAs (92 RLK, 9 NL, 19 TM-CC, 24 TX, 6 OTHER, 12 TN, 40 TNL, 28 RLP, 5 CNL, 4 RPW8, 4 RN and 2 CN) were found in clusters. When separate analyses were performed for each RGA type, 97 NLR genes (37 TNL, 23 TX, 12 TN, 9NL, 5 CNL, 5 OTHER, 4 RN and 2 CN), 81 RLKs, 22 RLP and 13 TM-CC genes were found in clusters, and no RPW8 was identified. In *S. arvensis*, the NLRs (96 genes; 28 TNL, 21 TX, 13 TN, 8 CNL, 15NL, 8 OTHER, 2 CN and 1RNL), RLK (105 genes), and RLP genes (27 genes) exhibited substantial clustering patterns within the genome, while RPW8 (4 genes) and TM-CC genes (8 genes) were less frequently found in such clusters. Furthermore, when we analysed all RGAs collectively, we identified 280 RGAs (122 RLK, 34 RLP, 31 TNL, 23 TX, 13 TN, 9 CNL, 17 TM-CC, 15 NL, 8 OTHER, 5 RPW8, 2 CN and 1 RNL) forming clusters.

In *S. alba*, separate analyses revealed that 24 NLR genes (9 TNL, 4 TX, 2 TN, 7 NL, 1 CN and 1 OTHER), 73 RLKs, 6 RLP, 12 TM-CC and 2 RPW8 genes were found in clusters; however, when all the RGAs were included in the analyses, 150 RGAs (84 RLK, 10 RLP, 22 TM-CC, 1 OTHER, 7 NL, 12 TNL, 3 RPW8, 6 TX, 3 TN, 1 CN and 1 RN) were found in clusters. The spatial distribution of these clustered RGAs across the chromosomes is illustrated in Supplementary Figure S5.

### 3.5. RGAs Sequence Pairwise Similarities

We also analysed pairwise similarities among different RGAs based on their gene sequences to understand the underlying relationships and patterns of similarity among these RGAs. The results of these analyses are represented as hierarchical clustering dendrograms, which revealed distinct groupings of genes. There were four clusters for *B. nigra* and five for *S. arvensis* and *S. alba*, each indicated by a unique colour (Figure S6a–c). In *B. nigra*, we identified four clusters: “green,” “orange,” “red,” and “purple,” each varying in the number of RGAs they harboured (Supplementary Figure S6a). The “green” cluster stood out as the largest, housing 1261 RGAs, with a predominant composition of 812 TM-LRR, 248 NLR, 9 RPW8, and 192 TM-CC genes. The “orange” cluster included 177 RGAs, with 83 TM-LRR, 53 NLR, and 41 TM-CC genes. In the “red” cluster, 143 RGAs were identified, consisting of 72 TM-LRR, 42 NLR, and 29 TM-CC genes. Lastly, the “purple” cluster contained 44 RGAs, comprising 18 TM-LRR, 14 NLR, 2 RPW8, and 10 TM-CC genes.

*S. arvensis* showed different arrangements of RGAs, with five clusters categorised by distinctive colours (Supplementary Figure S6b). The “brown” cluster was the largest, comprising 875 RGAs. It featured 549 TM-LRR, 188 NLR, 5 RPW8, and 133 TM-CC genes. The “purple” cluster contained 472 RGAs, with 280 TM-LRR, 112 NLR, and 80 TM-CC genes. The “green” cluster held 166 RGAs, consisting of 91 TM-LRR, 41 NLR, 3 RPW8, and 31 TM-CC genes. Meanwhile, the “red” cluster housed 80 RGAs, with 39 TM-LRR, 26 NLR, and 15 TM-CC genes. Finally, the “orange” cluster contained 32 RGAs, encompassing 14 TM-LRR, 7 NLR, 4 RPW8, and 7 TM-CC genes.

*S. alba* exhibited a similarly diverse pattern of RGAs as *S. arvensis*, with five “brown,” “purple,” “red,” “green,” and “orange” clusters (Supplementary Figure S6c). The “brown” cluster was the most substantial, comprising 886 RGAs. It comprised 644 TM-LRR, 91 NLR, 3 RPW8, and 148 TM-CC genes. In the “purple” cluster, 189 RGAs were identified, with 103 TM-LRR, 28 NLR, and 58 TM-CC genes. The “red” cluster held 114 RGAs, featuring 67 TM-LRR, 17 NLR, 1 RPW8, and 29 TM-CC genes. The “green” cluster contained 33 RGAs, including 8 TM-LRR, 17 NLR, 4 RPW8, and 4 TM-CC genes. Finally, the “orange” cluster housed 27 RGAs, encompassing 16 TM-LRR, 3 NLR, and 8 TM-CC genes.

### 3.6. RGA Homologue Identification

**Figure 2:**
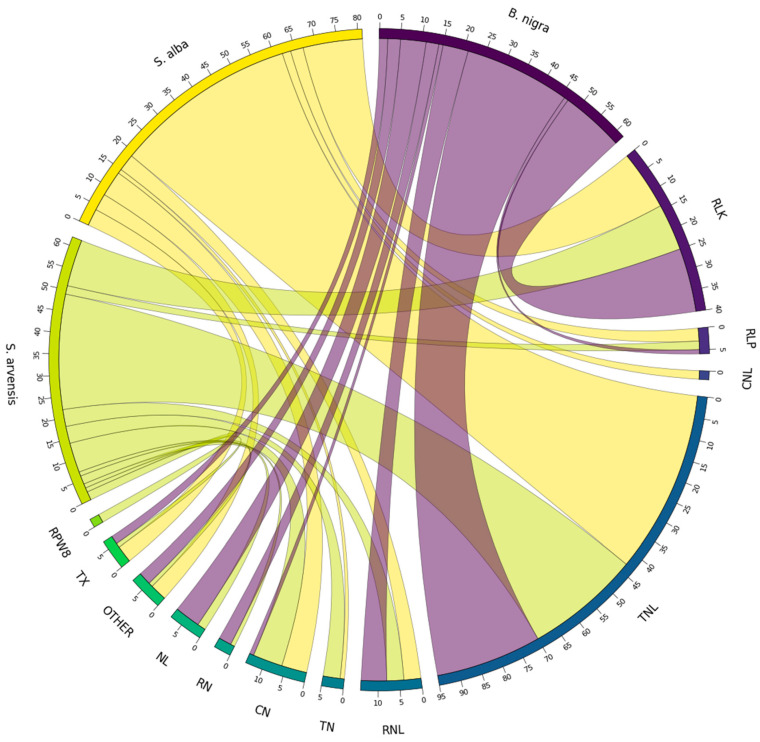
Distribution and count of cloned disease resistance gene homologues identified from 49 cloned *R* genes across the genomes of *B. nigra*, *S. arvensis*, and *S. alba*. The figure illustrates the presence of various resistance domains, including RLK, RLP, CNL, TNL, RNL, TN, CN, RN, NL, TX, OTHER, and RPW8, highlighting the number and distribution of CDRHs containing these domains within each species.

We utilised the protein sequences from 49 cloned *R* genes, which have been functionally validated to play a role in providing resistance against various diseases in the Brassicaceae. We used these sequences to identify homologous genes across *B. nigra*, *S. arvensis*, and *S. alba*. Our findings indicated that 18 cloned *R* genes had homologous genes among *B. nigra* RGAs. Among these, 13 cloned *R* genes had multiple homologues, with *At_WRR9* having the highest count at 10 (Supplementary Table S3). We identified a total of 62 CDRHs, which included 15 RLK, 25 TNL, 6 NL, 1 RLP, 3 RN, 6 RNL, 1 CN, 2 TX and 3 domains classified as OTHER (Figure 2). Additionally, we observed that 14 CDRHs in *B. nigra* were homologous to more than one of the cloned *R* genes (Supplementary Table S4).

There were 25 cloned *R* genes that had homologous genes in *S. arvensis* RGAs. Among these, 16 cloned *R* genes had multiple homologues, with *At_WRR9* having the highest count at 14 (Supplementary Table S3). We identified a total of 62 CDRHs, which included 11 RLK, 27 TNL, 2 NL, 2 RLP, 1 RN, 4 RNL, 4 TN, 7 CN, 1 TX, 2 RPW8, and 1 OTHER (Figure 2). Moreover, we found that 10 CDRHs in *S. arvensis* were homologous to more than one of the cloned *R* genes (Supplementary Table S4).

There were 23 cloned *R* genes that had homologous genes among *S. alba* RGAs. Of these, 16 cloned *R* genes had multiple homologues, with *At_RPP1* having the highest count at 9 (Supplementary Table S3). We identified 81 CDRHs, including 14 RLK, 43 TNL, 3 RLP, 4 RNL, 2 CNL, 1 TN, 6 CN, 4 TX, and 4 OTHER domains (Figure 2). Additionally, we found that 22 CDRHs in *S. alba* were homologous to more than one of the cloned *R* genes (Supplementary Table S4). The number and distribution of CDRHs containing resistance domains, including RLK, RLP, CNL, TNL, RNL, TN, CN, RN, NL, TX, OTHER and RPW8 in *B. nigra*, *S. arvensis* and *S. alba* genomes are shown in Figure 2.

**Figure 3:**
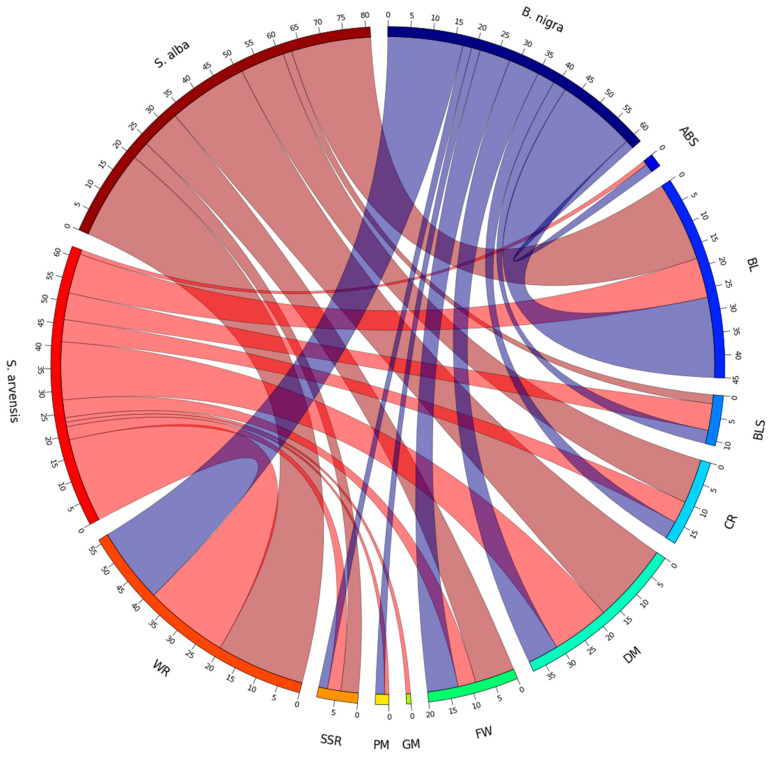
The number and distribution of cloned disease resistance gene homologues associated with resistance to various diseases, including Alternaria black spot (ABS), blackleg (BL), bacterial leaf spot (BLS), clubroot (CR), downy mildew (DM), Fusarium wilt (FW), grey mould (GM), powdery mildew (PM), Sclerotinia stem rot (SSR), and white rust (WR) in the genomes of B. nigra, S. arvensis, and S. alba. The data presented reflects the analysis of 205 CDRHs associated with the 49 cloned *R* genes, highlighting their contributions to disease resistance across these Brassicaceae species.

In total, 205 CDRHs were associated with cloned *R* genes that confer resistance to fungal and bacterial diseases, which impact species in the Brassicaceae family (Figure 3). Of these, 17 (associated with *At_RAC1*, *At_NGR1a,* and *At_WRR9* cloned *R* genes) and 18 (associated with *At_RLM1a*, *Bna_Rlm9/4/7* and *At_RLM1b* cloned *R* genes) CDRHs, conferring resistance to white rust and blackleg, respectively, were the highest numbers obtained in *B. nigra*. In *S. arvensis*, the highest numbers were 13 (associated with *At_RPP2a*, *At_RPP13*, *At_RPP8*, *At_NGR1b*, *At_RPP2b* and *At_RPP7* cloned *R* genes) and 19 (associated with *Bju_WRR1*, *At_NGR1a*, *At_RAC1* and *At_WRR9* cloned *R* genes) CDRHs, which were associated with downy mildew and white rust, respectively. For *S. alba*, the highest numbers were 18 (associated with *Bna_Rlm9/4/7*, *Bna_LepR3/Rlm2*, *At_RLM1a* and *At_RLM1b* cloned *R* genes), 18 (associated with *At_NGR1b*, *At_RPP8*, *At_RPP7*, *At_RPP1*, *At_RPP2b* and *At_RPP4* cloned *R* genes), and 20 (associated with *At_NGR1a*, *Bju_WRR1*, *At_WRR4a*, *At_WRR9* and *At_RAC1* cloned *R* genes) CDRHs, which were associated with resistance to blackleg, downy mildew, and white rust, respectively.

### 3.7. Non-RGA Homologues Identification

We performed BLASTp analyses involving the 49 cloned *R* genes against non-RGA genes in *B. nigra*, *S. arvensis*, and *S. alba*. The results revealed that in *B. nigra*, 16 out of the 49 cloned *R* genes exhibited homology with 36 non-RGA genes. Among these 16 cloned *R* genes, 8 showed homology with more than one non-RGA gene. Additionally, 7 non-RGA genes displayed homology with multiple cloned *R* genes (Supplementary Tables S3 and S4).

In *S. arvensis*, 14 of the 49 cloned *R* genes demonstrated homology with 23 non-RGA genes. Among the 14 cloned *R* genes, 7 displayed homology with more than one non-RGA gene. Similarly, 4 non-RGA genes exhibited homology with multiple cloned *R* genes (Supplementary Tables S3 and S4).

For *S. alba*, 9 out of the 49 cloned *R* genes showed homology with 18 non-RGA genes. Among these 9 cloned *R* genes, 2 were homologous with more than one non-RGA gene. Additionally, 1 non-RGA displayed homology with multiple cloned *R* genes (Supplementary Tables S3 and S4).

### 3.8. Co-Localisation of RLKs and RLPs to Reported Disease Resistance Loci

A total of 57 known QTL that are associated with disease resistance were identified in Brassicaceae crops from previous studies, which included 27 SSR, 19 BL, 3 CR, 4 BR, 3 WR and 1 HR QTL. Of these, 38 QTL were identified in *B. napus*, 10 in *B. juncea*, 8 in *B. oleracea* and one in *B. nigra*. Also, 30 QTL had overlaps with at least one other QTL (Supplementary Table S5). There were 591 out of 821 *B. nigra* RLKs and 118 out of 164 RLPs found to be colocalised in at least one of the 57 QTL regions. In *S. arvensis*, 810 out of 818 RLKs and 148 out of 155 RLPs reside within the QTL regions. For *S. alba*, 730 out of 734 RLKs and all 104 RLPs are located within at least one of the 57 QTL regions. This means that while all RLPs are present in at least one QTL, not all RLPs are necessarily found in every QTL region (Supplementary Table S6).

In *B. nigra*, we observed that BniB01g05710K (RLK) was present in 14 QTL, with the LMJR2 QTL exhibiting the highest number of RLKs (406) compared to other QTL. Additionally, BniB03g06770K (RLP) was identified in 13 QTLs, and once again, the LMJR2 QTL stood out with the highest count of RLPs (77) among the different QTLs (Supplementary Table S6).

For *S. arvensis*, both Sar07g06990R and Sar09g07950R (RLK) were identified in 14 QTLs, with the LMJR2 QTL containing the most RLKs (430) when compared to other QTLs. Moreover, we found 24 genes in 12 QTLs, and, as with RLPs, the LMJR2 QTL exhibited the highest number of RLPs (73) compared to other QTLs (Supplementary Table S6).

In *S. alba*, Sal02g07780L (RLK) was present in 14 QTLs, and, once again, the LMJR2 QTL demonstrated the highest number of RLKs (479) among all the QTLs. Additionally, Sal08g06760L (RLP) was identified in 13 QTLs, and as expected, the LMJR2 QTL contained the maximum number of RLPs (72) compared to other QTLs (Supplementary Table S6). In sum, the LMJR2 QTL, associated with blackleg resistance, contained the highest number of RLKs and RLPs across all three species compared to other QTLs in each species.

### 3.9. Phylogenetic Analysis

In the context of *B. nigra*, the phylogenetic analysis of the 49 cloned RGAs and QTL-mapped RLKs unveiled 23 distinct clusters. Among these, 15 clusters contained cloned *R* genes, with 2 specific groups exclusively composed of cloned RGAs. Group 1 (Indicated by an arrow; other groups are arranged in a clockwise direction) consisted of *At_RPP2a*, *At_RPP4*, *At_RLM1B*, *At_RPP1*, *Bra_Crr1a*, and *Bra_cRa/cRb*, while Group 2 included *At_WRR4b* (Supplementary Figure S7a). Additionally, in Group 3, 10 out of 16 RGAs were cloned RGAs. Concerning RLPs, out of 28 groups, 16 contained cloned *R* genes, with Groups 1 and 2 being the only clusters composed solely of cloned RGAs, containing 16 and 10 cloned RGAs, respectively (Supplementary Figure S7b).

In *S. arvensis*, the phylogenetic analysis of 49 cloned RGAs and QTL-mapped RLKs revealed 26 distinctive clusters, with 14 clusters containing cloned *R* genes. Notably, Groups 1 (*At_WRR4b*) and 2 (*At_RPP2a*, *At_RPP4*, *At_RLM1b*, *At_RPP1*, *Bra_Crr1a*, *Bra_cRa/cRb*) were the sole clusters, which were exclusively comprised of cloned RGAs (Supplementary Figure S7c). In terms of RLPs, among 45 groups, 21 contained cloned RGAs. Group 1 included 12 cloned RGAs, and Group 2 featured only 1 cloned RGA (Supplementary Figure S7d).

The analysis of *S. alba* identified 28 groups, with 16 containing cloned *R* genes and Group 1 being a unique cluster exclusively composed of cloned RGAs, specifically *At_WRR4b* and *At_WRR12*. Within Group 2, 8 out of 9 RGAs were cloned RGAs (Supplementary Figure S7e). Concerning RLPs, among 25 groups, 15 contained cloned *R* genes. Group 1, with 21 RGAs, impressively featured 16 cloned RGAs. Groups 2, 3, 4, and 7 contained only cloned RGAs, with 1, 3, 2, and 1 cloned RGAs, respectively (Supplementary Figure S7f).

## 4. Discussion

In this study, the selection of *B. nigra*, *S. alba*, and *S. arvensis* was strategic due to their significance in understanding plant resistance mechanisms, and it also offers the opportunity to identify novel sources of resistance that could enhance our knowledge and applications in plant breeding. These species were chosen based on their taxonomic closeness within the Brassicaceae family, making them valuable subjects for comparative genomic analysis. *B. nigra* and *Sinapis* species share a close genetic relationship. *S. arvensis* belongs to the same genus as *S. alba* and shares a more distant relationship with *B*. *nigra* [25]. Despite their taxonomic proximity, these species exhibit variations in their ecological niches and adaptation strategies, which could reflect diverse evolutionary paths in response to environmental pressures. Therefore, by investigating the genomes of these three species, we aim to elucidate commonalities and differences in their RGA composition, shedding light on the genetic basis of resistance mechanisms across closely related taxa and providing insights into plant defence strategies in Brassicaceae.

### 4.1. RGA Identification and Classification

Our analysis revealed 4499 candidate RGAs across the *B. nigra*, *S. arvensis* and *S. alba* genomes, with varying proportions in each species. This distribution of RGAs is an important finding as it reflects the diversity and complexity of the innate immune system in these Brassicaceae species. Similar patterns of RGA variation have been previously reported by Bayer et al. [44], who found that RGA candidates in *B. oleracea* are unevenly distributed across the genome, with the highest density in additional non-reference contigs, particularly for NBS-LRR genes. An uneven distribution of NLRs, RLPs and RLKs was observed between *B. juncea* chromosomes by Inturrisi et al. [34]. Classifying the identified RGAs into four main categories (RLKs, RLPs, NLRs and TM-CCs) further highlighted the genomic landscape of innate immunity. RLKs were the most prevalent class in all three species, constituting over 50% of the identified RGAs. This observation aligns with previous studies that have reported the significance of RLKs in pathogen recognition but also in signal transduction pathways in several species, including *Arabidopsis* [45,46], rice (*Oryza sativa*; [47,48,49], *Fragaria vesca* [50], *B. juncea* [51] and *B. napus* [52]. This can be attributed to the dual role of RLKs in signalling, which extends to various plant functions, including growth, development, and defence, as highlighted by Shiu et al. [47]. In contrast, NLR genes primarily focus on facilitating plant resistance responses, as emphasised by McHale et al. [53].

In our analysis of *S. alba*, we found no RLP genes on chromosome 03. This absence may indicate evolutionary or functional constraints on this chromosome. As mentioned by Tirnaz et al. [10], their results showed that a chromosome may be rich in one class of RGAs while containing few or no genes of another class. It also raises questions about the divergence of resistance mechanisms among Brassicaceae. Further research is needed to assess the implications of this finding for disease resistance in *S. alba* and to explore potential alternative resistance genes present in its genome.

We subdivided RLKs and RLPs based on their N-terminal domains, revealing distinct subgroups within each category. LRR-RLKs and LRR-RLPs were the most abundant subgroups, indicating the importance of leucine-rich repeats in these proteins. This abundance has been identified across numerous plant species. Examples include *Arabidopsis* [44], potato (*Solanum tuberosum*) [54], cotton (*Gossypium hirsutum*) [55], wheat (*Triticum aestivum*) [56] and rice [57]. The diversity of N-terminal domains within RLKs and RLPs suggests a wide range of pathogen recognition mechanisms, allowing these species to adapt to various pathogens and environmental conditions.

The results showed that the prevalence of atypical NLRs in all three species outnumbered typical NLRs. Atypical NLRs have been less characterised but are known to play a role in plant immunity. The exact numbers of typical and atypical NLR genes can vary between different *Brassica* species and even among cultivars within the same species [10]. Factors such as genome size, genome evolutionary history [10] and the biological cost of *R* gene copies [58] can influence the abundance and diversity of NLR genes. Many atypical NLRs form pairs, as seen in *Arabidopsis,* where the RPS4-RRS1 pair provides resistance to pathogens like *Ralstonia solanacearum* and *P. syringae*, with RPS4 being a typical TNL and RRS1 featuring an additional WRKY domain [59]. In addition to their role in NLR pairs, some atypical NLRs operate independently as “singletons” without a partner NLR [60]. This observation underscores the need for further investigation into these Brassicaceae species’ specific functions and mechanisms of atypical NLRs.

### 4.2. RGAs Distribution, Density and Chromosome Size Relationship

When examining the average number of RGAs per chromosome, *B. nigra* exhibited the highest density with an average of 201.62 RGAs per chromosome. *S. arvensis* followed with an average of 180.22 RGAs, while *S. alba* had a lower density with an average of 103.83 RGAs per chromosome. The lower average number of RGAs per chromosome in *S. alba* compared to *B. nigra* and *S. arvensis* can be attributed to its higher chromosome count (12), which likely distributes RGAs across a greater number of chromosomes, reducing the density per chromosome. In addition, differences in genome structure and evolutionary pressures could be a possible reason for the lack of a clear trend between RGA counts and genome size across the species [61]. Moreover, the genome size not only reflects the number of genes; it also includes non-coding regions, repetitive sequences [62], and transposable elements [63]. Therefore, a species with a larger genome may have more non-coding DNA that does not contribute to the RGA count, leading to a lower proportion of RGAs relative to its genome size.

In both *B. nigra* and *S. arvensis*, a general trend was observed where larger chromosomes tend to contain a higher number of RGAs. This trend implies that these species allocate a greater portion of their immune resources to larger chromosomes, possibly due to the need for robust pathogen recognition and defence on these chromosomes. However, there are noteworthy exceptions to this trend, where larger chromosomes harbour fewer RGAs. These exceptions suggest that other genetic or evolutionary factors play a role in determining the distribution of RGAs in specific chromosomes. Specific resistance loci or unique genomic architecture in these exceptions might account for this variation.

Generally, eukaryotes have more genes than prokaryotes, but their gene count does not strongly correlate with genome size. For instance, the yeast *Saccharomyces cerevisiae* has a 13.5 Mb genome with approximately 6400 genes, showing a relatively proportional relationship between genome size and gene number. In contrast, *Caenorhabditis elegans* has a much larger genome of 97 Mb but only about 18,000 genes. Therefore, genome size in eukaryotes does not necessarily correspond to the number of genes [64].

The comparison of RGA counts and genome size across these species indicates that genome size may not directly correlate with the total number of RGAs. Although *B. nigra* (515 Mb genome) and *S. arvensis* (450 Mb genome) have comparable RGA counts (1625), *S. alba*, with a slightly smaller genome (436 Mb), shows a substantially lower RGA count (1249). This suggests that factors other than genome size may influence RGA abundance across these species. As discussed by Tirnaz et al. [10], species with larger genome sizes do not necessarily harbour higher percentages of RGAs. For example, *Schrenkiella parvula* and *Capsella rubella* contained higher percentages of RGAs compared with the species with larger genomes, such as *B. napus* ‘Darmor*-bzh* V4’ and *B. juncea*. In the case of *S. alba*, a distinct pattern emerges wherein the larger chromosomes do not consistently exhibit a higher number of RGAs. This implies that in *S. alba*, factors other than chromosome size significantly influence the distribution of RGAs. The lack of a direct correlation between chromosome size and RGA numbers in *S. alba* may indicate the existence of species-specific defence strategies that do not conform to the general trend observed in the other two species. RGAs are often unevenly distributed across chromosomes. Researchers [65,66] created genetic maps for potatoes that included resistance traits. Their studies revealed that a significant number of NBS-LRR genes were located on chromosomes 4 and 11, accounting for approximately 15% of the mapped genes, whereas only 1% were found on chromosome 3. In *Medicago*, chromosomes 6 and 3 encoded about 34% and 40% of all TNLs, respectively [67].

The closer genetic relationship between *B. nigra* and *S. arvensis* compared to *S. alba*, as indicated by divergence time analyses [25], provides additional context. This closeness in evolutionary history likely contributes to the similarities in genomic structures and patterns observed between *B. nigra* and *S. arvensis*. It suggests that these species have shared a more recent common ancestor compared to *S. alba*, which could explain why they exhibit similar genomic features despite being distinct species. This complexity underscores the influence of various genetic and evolutionary mechanisms in shaping the distribution of RGAs and calls for further research to uncover the specific factors responsible for these observed patterns.

The examination of RGA distribution and density in the genomes of *B. nigra*, *S. arvensis*, and *S. alba* revealed interesting patterns and variations in the genomic organisation of these important immune response genes. Generally, RGAs were found to be more concentrated near the ends of each chromosome rather than at the centromeres, reflecting a common localisation pattern in these species. This distribution likely provides robust defence mechanisms at the genomic boundaries, where pathogens are more likely to encounter the plant’s defences. However, it is noteworthy that different types of RGAs were not evenly distributed along the chromosomes. This uneven distribution suggests that distinct categories of RGAs may have specific roles or requirements in defence strategies, leading to their non-uniform genomic placement. This finding resonates with existing literature, as evidenced by previous studies on *B. napus* and its progenitors *B. rapa* and *B. oleracea* genomes [31,68,69,70]. The similarity in distribution and density patterns of RGAs in the C sub-genome (*B. oleracea*) and the A sub-genome (*B. rapa*) with their counterparts in *B. napus* suggests conservation of RGAs during genome evolutionary events. This variation in RGA density among the species may reflect differences in their evolutionary and ecological contexts. The non-uniform distribution of different types of RGAs across the chromosomes suggests that each category may play distinct roles in the plant’s defence mechanisms. Similar results were documented by Tirnaz et al. [10] and Zhang et al. [70] who reported different RGA numbers in *C. rubella*, *Thellungiella salsuginea*, *A. lyrata*, *Arabidopsis*, and *B. rapa*.

### 4.3. Physical Clustering

The physical clustering of RGAs within the genome provides valuable insights into the genomic organisation and potential interactions of these immune response genes. Species such as *B. rapa*, *B. oleracea* [44], *B. napus* [71], and *B. juncea* [47] exhibit unique patterns of NLR gene duplications and clustering. These patterns are thought to be influenced by differing pathogen selection pressures among these species. In *B. nigra*, we observed varying degrees of clustering among RGAs, depending on the specific category. When all RGAs were considered collectively, 245 RGAs exhibited clustering and were located near each other. This clustering was particularly prominent in the NLR genes, with 97 NLRs forming clusters. This indicates a potential concentration of disease-resistance genes in specific genomic regions, which might be advantageous for coordinating defence responses.

When the analysis was separated by RGA category, we found that RLKs, RLPs, and TM-CC genes also displayed varying degrees of clustering. This indicates that different classes of RGAs may have specific patterns of physical organisation within the genome, possibly reflecting their roles and interactions in the plant’s immune response.

In the case of *S. arvensis*, a similar clustering pattern was observed. NLRs, RLKs, and RLPs showed substantial clustering. However, RPW8 and TM-CC genes exhibited less frequent clustering, indicating that these categories of RGAs may have different genomic distribution patterns.

*S. alba* exhibited distinct patterns of RGA clustering. When analysed separately, NLRs, RLKs, RLPs, TM-CC, and RPW8 genes were found in clusters, indicating the existence of spatial regions in the genome with higher concentrations of these defence-related genes. When all RGAs were collectively considered, 150 RGAs were found in clusters, again suggesting species-specific clustering patterns within the genome. *R* genes are commonly discovered in close physical proximity, forming clusters [46,72,73,74,75]. This observation implies that clustering provides evolutionary advantages to plants [76]. In addition, the spatial distribution of these clustered RGAs across the chromosomes can provide valuable information about potential hotspots for defence responses. Clustering RGAs within close genomic proximity may facilitate their coordinated action in response to pathogen attacks [77]. Therefore, clustering of these RGAs may help preserve diversity in disease resistance. When selection pressure arises, RGAs could function as accessory, helper, or sensor elements essential for disease resistance [78].

### 4.4. RGAs Sequence Pairwise Similarities

The analysis of pairwise similarities among RGAs based on their gene sequences reveals relationships and patterns of similarity within these crucial components of plant immune systems. Multiple clusters within these species emphasise the complexity and diversity of the immune response genes [79]. These clusters likely represent specific genomic regions or pathways dedicated to different aspects of the defence mechanism, such as pathogen recognition, signal transduction, or effector molecules [80]. The variation in RGA composition and the distinct clustering patterns within each species underscore their unique genomic organisation and adaptation to specific environmental challenges.

### 4.5. RGA and Non-RGA Homologues Identification

Identifying homologous genes among RGAs and non-RGAs is critical to understanding the genetic basis of disease resistance. By comparing the cloned *R* genes with the genomes of *B. nigra*, *S. arvensis*, and *S. alba*, we aimed to identify homologous genes that could play roles in disease resistance. The results revealed several interesting patterns of homology between cloned *R* genes and RGAs, providing valuable insights into the genetic diversity of resistance mechanisms within these species.

In *B. nigra*, we found that 18 cloned *R* genes had homologous genes among the RGAs. These homologues were distributed across various RGA categories, indicating a diverse set of resistance mechanisms present in the genome. Some cloned *R* genes had multiple homologues, emphasising the complexity and redundancy of disease resistance mechanisms within *B. nigra*. *S. arvensis* exhibited similar patterns of homology, with 25 cloned *R* genes having homologous genes among its RGAs. Again, this diversity was reflected in multiple homologues for several cloned *R* genes, highlighting the complexity of resistance mechanisms in *S. arvensis*. In *S. alba*, 23 cloned *R* genes showed homology with its RGAs. This species also demonstrated multiple homologues for specific cloned *R* genes, suggesting various disease resistance mechanisms. The presence of homologous genes for cloned *R* genes across these species underscores the evolutionary conservation of specific resistance mechanisms within the Brassicaceae family. The relatively low number of homologous genes among the RGAs in these species may be attributed to their diploid nature. Previous literature suggests that polyploidisation, as seen in *Brassica* species, typically results in a larger number of total cloned RGAs compared to diploid species [30,81]. Furthermore, previous studies have indicated that polyploid *Brassica* species tend to exhibit higher counts of genes, including RGAs, and genes related to glucosinolate, in comparison to their diploid progenitor species [10,81]. These findings may aid in understanding the genetic basis of disease resistance and provide insights into potential targets for crop improvement and breeding strategies. The diversity of homologues within different RGA categories suggests a multifaceted approach to disease resistance, where various genes and pathways protect these plants from a wide range of pathogens.

Additionally, our study explored homology between cloned *R* genes and non-RGA genes, revealing that several cloned *R* genes exhibited homology with non-RGA genes in these species. The non-RGA genes may have functions related to resistance or contain partial RGA domain structures (lacking some key domains). For instance, Cantila et al. [24] found a non-RGA, AT3G20590, which resembled a non-specific disease resistance protein [82] and clustered with the *At_NDR1* gene. Also, a non-RGA, B08g104510.1, was identified as a mitogen-activated protein kinase [32], clustered with the NLRs Bo8g104700.1, Bo8g104710.1, and Bo8g104730.1. They have also found that a non-RGA, BnaA02g24430D, which includes an LRR domain [31], clustered with BnaA02g24440D, an LRR-RLP. This highlights the potential for broader genetic interactions between disease-resistance genes and other genetic elements within the genomes of *B. nigra*, *S. arvensis*, and *S. alba*.

### 4.6. Co-Localisation of RLKs and RLPs to Reported Disease Resistance Loci

We specifically focused on RLKs and RLPs in our study of co-localisation with reported disease resistance loci in *Brassica* crops due to their established importance as key components of plant immune signalling pathways [83]. RLKs and RLPs are pivotal in recognising PAMPs and initiating defence responses [84]. While other RGAs, including NLRs, also play significant roles in disease resistance across a wide range of *Brassicas*, RLKs and RLPs are particularly notable in immunity and have been extensively researched in the context of disease resistance mechanisms. NLR proteins have a conserved modular structure crucial for their role in immunity [85]. In plants, NLRs play a key role in triggering programmed cell death in response to pathogens [86], with their N-terminal signalling domains, central NB-ARC domains, and C-terminal leucine-rich repeats (LRRs) working together to initiate immune responses [87].

While the majority of RGAs underlying the QTL were found to be NLR genes, which are crucial in plant disease resistance [53,88], many previously cloned resistance genes for white rust and blackleg resistance were identified as RLKs and RLPs. For example, the blackleg *R* genes *Rlm2* and *LepR3*, cloned from *Brassica* species, encode RLPs with extracellular leucine-rich repeat domains located on chromosome A10 [89,90]. *LepR3*, annotated as Bra008930 in *B. rapa*, and *Rlm2* cloned from *B. napus*, have a motif structure (predicted via InterProScan) that includes an N-terminal signal peptide, an eLRR region, a transmembrane motif, and a cytoplasmic C-terminal region [89,90]. *LepR3* and *Rlm2* are allelic and encode membrane-bound LRR-RLPs. *Rlm2* was shown to interact with AtSOBIR1, an LRR-RLK gene [90], while *LepR3* was demonstrated to interact with BnSOBIR1, an RLK in *B. napus* [91]. It is likely that the initial study was conducted with *Arabidopsis*, and subsequent identification of the *B. napus* copy was possible after the *B. napus* genome was sequenced. These findings underscore the importance of including RLKs and RLPs, in addition to NLRs, for identifying functional *R* genes.

Our results highlight the co-localisation of disease resistance-related genes, specifically RLKs and RLPs, within QTL in various Brassicaceae crops, which makes them important targets for plant breeding. Plants use RLKs and RLPs as PRRs to sense their apoplastic environment and identify MAMPs or DAMPs, signalling potential threats and triggering PTI [92]. For example, the cucumber genome contains 178–192 RLKs and 42–56 RLPs, with some co-localised within the QTL region for downy mildew resistance [93]. This suggests that downy mildew resistance genes may encode RLPs or RLKs, providing cultivar-specific resistance similar to NLRs. Inturrisi et al. [34] mapped nine genomic regions related to disease resistance in *B. juncea,* where the regions carry 14 RLPs and 115 RLKs. They found that RLKs were the most prevalent in all QTL, with most QTL containing more NLRs than RLPs, except for LMJR2 on chromosome B08, which had 2 NLRs and 6 RLPs.

The co-localisation of RLK and RLP genes within previously identified QTL regions suggests a significant role for these genes in the genetic factors contributing to disease resistance in *Brassica* species. However, it remains to be determined whether these co-localisation patterns reflect functional conservation across species or represent species-specific diversification. Although the observed co-localisation of RLK and RLP genes within previously identified QTL regions suggests potential functional roles in traits of interest, future studies involving comparative expression analyses, functional validation, and evolutionary investigations will be essential to clarify the evolutionary dynamics and functional relevance of these genes in crop defence mechanisms.

### 4.7. Phylogenetic Analysis

The phylogenetic analysis results shed light on the genetic diversity and organisation of *R* genes in *B. nigra*, *S. arvensis* and *S. alba*. The phylogenetic analysis revealed several clusters of RGAs. These findings are significant because they provide insights into the genetic organisation of *R* genes in these three closely related plant species. The presence of unique clusters exclusively composed of cloned RGAs and the varying distribution of cloned RGAs among different groups suggest a complex interplay between *R* genes in these species.

## 5. Conclusions

In this study, we conducted a comprehensive identification, classification, and distributional analysis of resistance gene analogues (RGAs) in the genomes of *B. nigra*, *S. arvensis*, and *S. alba*. By applying the RGAugury pipeline, we identified and classified RGAs into major categories, revealing a rich diversity across the species. The analysis revealed that different RGA classes exhibited varying levels of prevalence and distribution, with distinct patterns observed in relation to chromosome size. The study highlighted clustering among RGA types, suggesting interesting relationships within the gene families. The clustering of RGAs on chromosomes may facilitate coordinated gene expression, enabling a robust immune response in plants during pathogen attacks. Furthermore, we identified homologs of known *R* genes, underscoring similarities and conservation cues that might contribute to understanding resistance mechanisms. Also, regions associated with resistance traits revealed a significant presence of specific RGA classes, indicating potential hotspots for gene co-localisation. Phylogenetic analysis further illustrated the evolutionary dynamics and possible functional adaptations of these resistance genes across the studied species that may play specialised roles in pathogen recognition and defence. These genetic complexities likely enhance disease resistance by providing multiple defensive strategies against pathogens. These findings offer valuable insights into plant immunity and have significant implications for future crop breeding strategies. By harnessing the genetic diversity of RGAs in *B. nigra*, *S. arvensis*, and *S. alba*, breeders could develop cultivars with improved disease resistance, contributing to sustainable agricultural practices.

In conclusion, our study underscores the rich genetic diversity and complex organisation of resistance genes within *B. nigra*, *S. arvensis*, and *S. alba*. This information is crucial for developing new crop varieties with enhanced disease resistance, reducing the reliance on chemical treatments, and promoting sustainable farming practices.

### Limitations and Future Directions

While this study provides a comprehensive overview of RGAs in *B. nigra*, *S. arvensis*, and *S. alba*, there remain opportunities for further exploration and refinement. The analyses were primarily based on in silico predictions without experimental validation, and the functional roles of identified RGAs remain to be confirmed. Also, the study did not investigate gene expression or epigenetic regulation, which could provide deeper insight into resistance mechanisms. Future studies should incorporate orthology and synteny analyses between species, particularly when comparing QTL regions to strengthen cross-species comparisons. This approach should also include transcriptomic and functional validation studies, such as gene expression profiling under pathogen challenge and CRISPR-based gene knockouts, to determine the roles of key RGAs. Despite these limitations, this study delivers new knowledge by cataloguing the diversity and phylogenetic relationships of RGAs across three important Brassicaceae species. The identification of clustered RGAs within disease-resistance QTLs, particularly RLKs and RLPs, provides a genomic framework for targeted introgression of resistance loci into cultivated crops. These findings can directly inform marker-assisted selection and gene pyramiding strategies in Brassica breeding programmes, ultimately aiding in the development of disease-resilient cultivars and supporting more sustainable agricultural systems.

## Figures and Tables

**Table 1:** Reported disease resistance quantitative trait loci (QTL) of blackleg (BL), black rot (BR), clubroot (CR), hypocotyl rot (HR), Sclerotinia stem rot (SSR) and white rust (WR) in *Brassica napus* (Bna), *Brassica juncea* (Bju) and *Brassica oleracea* (Bol).

QTL	Species	Disease	Chromosome (QTL Coordinates)	Reference
A7	Bna	BL	A08 (9,926,520–14,644,781)	[33]
A8.dy09	Bna	BL	A08 (9,514,104–15,735,553)	
A9.dy05	Bna	BL	A09 (5,371,869–20,078,473)	
C6.dy13	Bna	BL	C06 (13,138,327–20,245,793)	
C8	Bna	BL	C08 (23,938,650–35,120,753)	
CRQTL-GN_2	Bna	CR	C03 (1,185,066–2,835,468)	
Dw12	Bna	SSR	C02 (3,710,868–6,707,092)	
Dw16	Bna	SSR	C06 (28,554,990–35,465,622)	
Dw3	Bna	SSR	A03 (15,547,362–16,064,878)	
LepR1	Bna	BL	A02 (11,756,829–18,651,329)	
LRA9-1	Bna	SSR	A09 (22,050,676–23,688,607)	
LRA9-2	Bna	SSR	A09 (22,050,676–22,586,868)	
LRC5	Bna	SSR	C04 (3,065,391–7,930,616)	
qFR10-1	Bna	SSR	C02 (1,027,454–3,174,971)	
qFR10-2	Bna	SSR	C02 (1,099,275–6,816,020)	
qFR11-1	Bna	SSR	A09 (29,166,099–29,361,292)	
qFR11-3	Bna	SSR	C02 (1,027,454–3,174,971)	
qSR10-1	Bna	SSR	A02 (1,610,851–7,705,833)	
qSR10-2	Bna	SSR	A03 (3,131,828–6,786,833)	
qSR10-3	Bna	SSR	C02 (1,027,454–3,953,336)	
qSR11-1	Bna	SSR	A09 (27,128,147–28,071,597)	
qSR11-2	Bna	SSR	C02 (1,027,454–3,953,336)	
Rlm3	Bna	BL	A07 (15,120,000–16,290,000)	
SCR-C6	Bna	CR	C06 (25,090,000–26,220,000)	
Sll14a	Bna	SSR	C04 (396,764–9,418,160)	
Sll14b	Bna	SSR	C04 (11,691,778–28,720,453)	
Sll2	Bna	SSR	A02 (32,458–3,454,175)	
SRA1	Bna	SSR	A01 (12,444,829–19,857,639)	
SRA2-1	Bna	SSR	A02 (16,670,964–20,474,897)	
SRA2-2	Bna	SSR	A02 (21,084,362–24,719,312)	
SRA6	Bna	SSR	A06 (20,965,425–23,324,273)	
SRA8	Bna	SSR	A08 (7,467,851–8,338,138)	
SRA9-1	Bna	SSR	A09 (22,586,748–26,573,318)	
SRC6-1	Bna	SSR	C06 (30,278,840–34,585,422)	
SRC6-2	Bna	SSR	C06 (30,278,840–34,585,422)	
SRC7	Bna	SSR	C07 (29,634,609–31,761,057)	
TS A09	Bna	BL	A09 (24,341,296–25,991,630)	
AcB1-A4.1	Bju	WR	A04 (9,446,467–11,808,704)	[34]
AcB1-A5.1	Bju	WR	A05 (3,795,221–6,894,070)	
AcB1-A5.1	Bju	WR	B06 (4,226,533–7,156,115)	
BjCHI1	Bju	HR	A03 (9,353,574–21,355,565)	
LMJR1	Bju	BL	B03 (498,805–10,675,185)	
LMJR2	Bju	BL	B08 (1–21,282,056)	
PhR2	Bju	BL	A08 (21,485,767–24,843,799)	
PhR2	Bju	BL	B03 (1,554,162–4,778,538)	
BRQTL-C1_1	Bol	BR	C01 (14,884,502–16,579,946)	[35]
BRQTL-C1_2	Bol	BR	C01 (18,227,386–37,119,290)	
BRQTL-C3	Bol	BR	C03 (19,714,632–22,846,644)	
BRQTL-C6	Bol	BR	C06 (7,423,787–10,466,894)	
LepR1	Bol	BL	C02 (23,420,917–39,667,823)	[36]
LepR2	Bol	BL	C09 (36,661,274–41,215,564)	[37]
LepR4	Bol	BL	C03 (35,912,191–49,368,477)	[38]
Rlm1	Bol	BL	C06 (20,455,085–36,165,661)	[39]
Rcr6	Bni	CR	B03 (6,100,000–6,600,000)	[40]
Rlm6	Bju	BL	A07 (28,140,000–28,631,000)	[41]
Rlm6	Bju	BL	B04 (19,804,000–22,303,000)	
Rlm13	Bna	BL	C03 (2,573,230–5,711,418)	[42]

**Table 2:** Number of different RGAs identified in *B. nigra, S. arvensis* and *S. alba*.

Species	Position (Mbp)	RLK	LRR-RLK	LysM-RLK	Other Receptor	RLP	LRR-RLP	LysM-RLP	Other Receptor	TM-CC	TNL	CNL	RNL	TX	TN	NL	RN	CN	OTHER	RPW8	Total	RGA/Mbp
*B. nigra*	Chr01 (54.73)	74	28	4	42	10	10	0	0	24	9	0	0	4	3	3	0	0	2	2	**131**	2.39
	Chr02 (73.74)	147	57	0	90	23	23	0	0	53	20	6	3	16	5	8	2	2	4	4	**293**	3.97
	Chr03 (59.02)	117	44	0	73	17	17	0	0	40	22	8	0	3	9	4	2	2	6	0	**230**	3.89
	Chr04 (51.41)	87	38	1	48	28	27	1	0	30	35	13	0	23	5	5	2	4	4	1	**237**	4.60
	Chr05 (67.89)	132	47	0	85	29	28	1	0	29	7	3	1	12	6	11	2	1	3	1	**237**	3.49
	Chr06 (61.87)	83	37	0	46	22	22	0	0	30	3	4	2	4	0	2	1	1	2	0	**154**	2.48
	Chr07 (59.87)	61	33	1	27	17	17	0	0	32	4	1	3	3	1	1	0	3	2	0	**128**	2.13
	Chr08 (71.98)	118	39	0	79	15	15	0	0	30	19	2	0	3	1	3	0	2	7	3	**203**	2.82
	Contig 005	1	1	0	0	0	0	0	0	0	0	0	0	0	0	0	0	0	0	0	**1**	-
	Contig 011	0	0	0	0	1	1	0	0	0	0	0	0	0	0	0	0	0	0	0	**1**	-
	Contig 013	0	0	0	0	0	0	0	0	1	0	0	0	0	0	0	0	0	0	0	**1**	-
	Contig 032	0	0	0	0	1	1	0	0	0	0	0	0	0	0	0	0	0	0	0	**1**	-
	Contig 041	0	0	0	0	0	0	0	0	1	0	0	0	0	0	0	0	0	0	0	**1**	-
	Contig 048	0	0	0	0	0	0	0	0	0	0	0	0	1	0	0	0	0	0	0	**1**	-
	Contig 067	0	0	0	0	0	0	0	0	1	0	0	0	0	0	0	0	0	0	0	**1**	-
	Contig 158	0	0	0	0	1	1	0	0	0	0	0	0	0	0	0	0	0	0	0	**1**	-
	Contig 193	0	0	0	0	0	0	0	0	0	0	0	0	1	0	0	0	0	0	0	**1**	-
	Contig 296	1	1	0	0	0	0	0	0	0	0	0	0	0	0	0	0	0	0	0	**1**	-
	Contig 323	0	0	0	0	0	0	0	0	1	0	0	0	0	0	0	0	0	0	0	**1**	-
	Contig 353	0	0	0	0	0	0	0	0	0	0	0	0	1	0	0	0	0	0	0	**1**	-
	**Total**	**821**	**325**	**6**	**490**	**164**	**162**	**2**	**0**	**272**	**119**	**37**	**9**	**71**	**30**	**37**	**9**	**15**	**30**	**11**	**1625**	
*S. arvensis*	Chr01 (46.40)	75	23	2	50	13	13	0	0	20	10	1	0	7	1	0	0	0	1	1	**129**	2.78
	Chr02 (43.41)	124	45	1	78	15	15	0	0	38	14	4	1	13	4	3	2	1	3	0	**222**	5.11
	Chr03 (37.28)	65	28	1	36	23	23	0	0	26	11	5	0	5	8	0	0	0	4	0	**147**	3.51
	Chr04 (47.78)	91	34	0	57	13	13	0	0	28	26	0	0	5	6	3	1	2	3	4	**182**	3.80
	Chr05 (52.17)	68	35	0	33	16	16	0	0	31	39	9	5	28	7	15	1	13	3	1	**236**	4.52
	Chr06 (49.85)	125	39	0	86	27	26	1	0	33	14	3	0	4	5	3	1	0	3	2	**220**	4.41
	Chr07 (49.15)	86	38	0	48	16	16	0	0	30	3	9	2	3	3	6	1	1	2	0	**162**	3.29
	Chr08 (42.09)	104	33	1	70	11	11	0	0	33	8	5	0	1	1	3	2	3	2	1	**174**	4.13
	Chr09 (36.73)	80	25	0	55	20	19	1	0	25	7	5	0	3	4	0	0	2	1	3	**150**	4.08
	Contig 357	0	0	0	0	0	0	0	0	1	0	0	0	0	0	0	0	0	0	0	**1**	-
	Contig 358	0	0	0	0	1	1	0	0	0	0	0	0	0	0	0	0	0	0	0	**1**	-
	Contig 452	0	0	0	0	0	0	0	0	1	0	0	0	0	0	0	0	0	0	0	**1**	-
	**Total**	**818**	**300**	**5**	**513**	**155**	**153**	**2**	**0**	**266**	**132**	**41**	**8**	**69**	**39**	**33**	**8**	**22**	**22**	**12**	**1625**	
*S. alba*	Chr01 (57.15)	55	19	2	34	7	7	0	0	17	3	0	0	2	1	1	0	0	0	0	**86**	1.50
	Chr02 (31.68)	55	28	1	26	11	11	0	0	35	2	2	0	0	1	0	0	1	1	0	**108**	3.40
	Chr03 (31.00)	78	21	0	57	0	0	0	0	15	3	0	2	0	1	1	1	0	0	0	**101**	3.25
	Chr04 (32.85)	62	31	0	31	2	2	0	0	33	6	1	0	11	1	4	2	1	2	3	**128**	3.89
	Chr05 (28.50)	60	20	1	39	10	9	1	0	12	6	2	0	2	0	0	1	1	0	0	**94**	3.29
	Chr06 (38.87)	60	22	1	37	7	7	0	0	20	7	1	0	6	4	3	1	1	0	1	**111**	2.85
	Chr07 (29.24)	65	27	0	38	9	9	0	0	23	2	1	1	2	1	0	0	0	1	2	**107**	3.65
	Chr08 (32.51)	48	19	1	28	14	13	1	0	16	2	4	0	4	1	1	0	0	0	0	**90**	2.76
	Chr09 (44.42)	79	31	0	48	13	12	1	0	18	4	2	0	2	0	1	1	2	1	1	**124**	2.79
	Chr10 (28.29)	60	24	0	36	8	8	0	0	18	7	0	1	6	2	3	0	2	1	0	**108**	3.81
	Chr11 (30.85)	48	23	0	25	7	7	0	0	23	3	2	0	0	0	0	1	2	2	1	**89**	2.88
	Chr12 (30.69)	62	22	0	40	15	15	0	0	17	2	0	0	2	1	0	0	1	0	0	**100**	3.25
	Contig 191	0	0	0	0	1	1	0	0	0	0	0	0	0	0	0	0	0	0	0	**1**	-
	Contig 373	1	0	0	1	0	0	0	0	0	0	0	0	0	0	0	0	0	0	0	**1**	-
	Contig 456	1	0	0	1	0	0	0	0	0	0	0	0	0	0	0	0	0	0	0	**1**	-
	**Total**	**734**	**287**	**6**	**441**	**104**	**101**	**3**	**0**	**247**	**47**	**15**	**4**	**37**	**13**	**14**	**7**	**11**	**8**	**8**	**1249**	

## Data Availability

The data that support the findings of this study are openly available in figshare.

## Supplementary Materials

The following supporting information can be downloaded at: https://www.mdpi.com/article/10.3390/genes16080849/s1, Figure S1: Distribution and overlap of 57 QTL associated with disease resistance in *Brassica* crops. QTL are mapped across 21 chromosomes and confer resistance to blackleg (BL), black rot (BR), clubroot (CR), hypocotyl rot (HR), Sclerotinia stem rot (SSR), and white rust (WR) (see Table 1 for details). Overlapping regions among these QTL are illustrated. Figure S2: The percentage of RGAs on each chromosome and contigs. **a**: *B. nigra*, **b**: *S. arvensis* and **c**: *S. alba*. The coloured bars show the percentage of RGAs in chromosomes and contigs. Figure S3: Relationship between chromosome and genome size with the number of RGAs. Figure S4: **a**, **b** and **c**: RGAs positions on the chromosomes. Outer to inner, RLK, RLP, TM-CC, RPW8 and NLR. **d, e** and **f:** RGAs size. Grey lines show average size. Figure S5: Spatial distribution of clustered RGAs across the *B. nigra* (**a**), *S. arvensis* (**b**) and *S. alba* (**c**) chromosomes. Clusters were defined as groups with a threshold distance of 10 kb. Figure S6: The hierarchical clustering dendrogram visually represents the relationships among the RGA sequences. In this dendrogram, genes are clustered based on their sequence similarities, with closer branches indicating higher similarity. The colour-coded branches emphasise the division of the RGAs into four (*B. nigra*) and five (*S. arvensis* and *S. alba*) discernible clusters. Figure S7: Phylogenetic analysis of 49 cloned RGAs, RLKs, and RLPs found within QTLs associated with disease resistance in *Brassica* crops. **a**, **b** and **c**: RLKs and cloned RGAs in *B. nigra*, *S. arvensis* and *S. alba*, respectively. **d, e** and **f:** RLPs and cloned RGAs in *B. nigra*, *S. arvensis* and *S. alba*, respectively. Group 1 is indicated by an arrow to distinguish it from the other groups, which are arranged in a clockwise direction. Table S1: The 49 cloned R genes from Arabidopsis thaliana (At), Brassica juncea (Bju), Brassica napus (Bna) and Brassica rapa (Bra) used for homology searches. Table S2: Full chromosome pairwise comparison for B. nigra, S. arvensis, and S. alba showing whether the larger chromosome has more RGAs. Table S3: Cloned R genes and their corresponding RGA and non-RGA homologs across B. nigra, S. arvensis and S. alba genomes. Table S4: RGA and non-RGA, homologous to more than one of the cloned R genes across B. nigra, S. arvensis and S. alba genomes. Table S5: QTL overlapping with at least one other QTL. Table S6: The RLKs and RLPs located outside (O) or within (W) QTL regions in three species.

## References

[1] Mohd Saad N.S., Severn-Ellis A.A., Pradhan A., Edwards D., Batley J. (2021). Genomics armed with diversity leads the way in *Brassica* improvement in a changing global environment. Front. Genet..

[2] Dwivedi S.L., Scheben A., Edwards D., Spillane C., Ortiz R. (2017). Assessing and exploiting functional diversity in germplasm pools to enhance abiotic stress adaptation and yield in cereals and food legumes. Front. Plant Sci..

[3] Greer S.F., Surendran A., Grant M., Lillywhite R. (2023). The current status, challenges, and future perspectives for managing diseases of *Brassicas*. Front. Microbiol..

[4] Bhattacharya I., Dutta S., Mondal S., Mondal B. (2014). Clubroot disease on *Brassica* crops in India. Can. J. Plant Pathol..

[5] Barbetti M.J., Li C.X., Banga S.S., Banga S.K., Singh D., Sandhu P.S., Singh R., Liu S.Y., You M.P. (2015). New host resistances in *Brassica napus* and *Brassica juncea* from Australia, China and India: Key to managing *Sclerotinia* stem rot (*Sclerotinia sclerotiorum*) without fungicides. Crop Prot..

[6] Chattopadhyay C., Kolte S.J., Waliyar F. (2015). Diseases of Edible Oilseed Crops.

[7] Barbetti M.J., Li C.X., You M.P., Singh D., Agnihotri A., Banga S.K., Sandhu P.S., Singh R., Banga S.S. (2016). Valuable new leaf or inflorescence resistances ensure improved management of white rust (*Albugo candida*) in mustard (*Brassica juncea*) crops. J. Phytopathol..

[8] Jones J.D., Dangl J.L. (2006). The plant immune system. Nature.

[9] Sekhwal M.K., Li P., Lam I., Wang X., Cloutier S., You F.M. (2015). Disease resistance gene analogs (RGAs) in plants. Int. J. Mol. Sci..

[10] Tirnaz S., Bayer P., Inturrisi F., Neik T., Yang H., Dolatabadian A., Zhang F., Severn-Ellis A., Patel D., Pradhan A. (2020). Resistance gene analogs in the Brassicaceae: Identification, characterisation, distribution and evolution. Plant Physiol..

[11] Cui F., Wu S., Sun W., Coaker G., Kunkel B., He P., Shan L. (2013). The Pseudomonas syringae type III effector AvrRpt2 promotes pathogen virulence via stimulating *Arabidopsis* auxin/indole acetic acid protein turnover. Plant Physiol..

[12] Chisholm S.T., Coaker G., Day B., Staskawicz B.J. (2006). Host—Microbe interactions: Shaping the evolution of the plant immune response. Cell.

[13] Zhao Q., Bao J., Li H., Hu W., Kong Y., Zhong Y., Fu Q., Xu G., Liu F., Jiao X. (2024). Structural and biochemical basis of FLS2-mediated signal activation and transduction in rice. Plant Commun..

[14] Afzal A.J., Wood A.J., Lightfoot D.A. (2008). Plant receptor—Like serine threonine kinases: Roles in signaling and plant defense. Mol. Plant–Microbe Interact..

[15] Jeong S., Trotochaud A.E., Clark S.E. (1999). The *Arabidopsis CLAVATA2* gene encodes a receptor-like protein required for the stability of the *CLAVATA1* receptor-like kinase. Plant Cell.

[16] Le Roux C., Huet G., Jauneau A., Camborde L., Trémousaygue D., Kraut A., Zhou B., Levaillant M., Adachi H., Yoshioka H. (2015). A receptor pair with an integrated decoy converts pathogen disabling of transcription factors to immunity. Cell.

[17] Ravensdale M., Bernoux M., Ve T., Kobe B., Thrall P.H., Ellis J.G., Dodds P.N. (2012). Intramolecular interaction influences binding of the flax L5 and L6 resistance proteins to their AvrL567 ligands. PLoS Pathog..

[18] Nadeau J.A., Sack F.D. (2002). Control of stomatal distribution on the *Arabidopsis* leaf surface. Science.

[19] Quezada-Martinez D., Addo Nyarko C.P., Schiessl S.V., Mason A.S. (2021). Using wild relatives and related species to build climate resilience in *Brassica* crops. Theor. Appl. Genet..

[20] Hasan M.J., Strelkov S.E., Howard R.J., Rahman H. (2012). Screening of Brassica germplasm for resistance to *Plasmodiophora brassicae* pathotypes prevalent in Canada for broadening diversity in clubroot resistance. Can. J. Plant Sci..

[21] Chu M., Yu F., Falk K., Liu X., Zhang X., Chang A., Peng G. (2013). Identification of the clubroot resistance gene Rpb1 and introgression of the resistance gene into canola breeding lines using a marker-assisted selection approach. Acta Hortic..

[22] Peng G., Falk K.C., Gugel R.K., Franke C., Yu F., James B., Strelkov S.E., Hwang S.-F., McGregor L. (2014). Sources of resistance to *Plasmodiophora brassicae* (clubroot) pathotypes virulent on canola. Can. J. Plant Pathol..

[23] Chevre A.M., Eber F., This P., Barret P., Tanguy X., Brun H., Delseny M., Renard M. (1996). Characterization of *Brassica nigra* chromosomes and of blackleg resistance in *B. napus*–*B. nigra* addition lines. Plant Breed..

[24] Cantila A.Y., Thomas W.J.W., Bayer P.E., Edwards D., Batley J. (2024). In silico prediction and analysis of transmembrane-coiled-coil resistance gene analogues in 27 Brassicaceae species. Plant Pathol..

[25] Yang T., Cai B., Jia Z., Wang Y., Wang J., King G.J., Ge X., Li Z. (2023). *Sinapis* genomes provide insights into whole-genome triplication and divergence patterns within tribe Brassiceae. Plant J..

[26] Li P., Quan X., Jia G., Xiao J., Cloutier S., You F.M. (2016). RGAugury: A pipeline for genome-wide prediction of resistance gene analogs (RGAs) in plants. BMC Genom..

[27] Anand L., Rodriguez Lopez C.M. (2022). ChromoMap: An R package for interactive visualization of multi-omics data and annotation of chromosomes. BMC Bioinform..

[28] Cantila A.Y., Neik T.X., Tirnaz S., Thomas W.J.W., Bayer P.E., Edwards D., Batley J. (2022). Mining of cloned disease resistance gene homologs (CDRHs) in *Brassica* species and *Arabidopsis thaliana*. Biology.

[29] Wu T., Al-Mamun H.A., Edwards D., Batley J., Dolatabadian A. (2024). Genome-wide identification and prediction of disease resistance genes in *Hirschfeldia incana*. Agric. Commun..

[30] Yang J., Liu D., Wang X., Ji C., Cheng F., Liu B., Hu Z., Chen S., Pental D., Ju Y. (2016). The genome sequence of allopolyploid *Brassica juncea* and analysis of differential homeolog gene expression influencing selection. Nat. Genet..

[31] Chalhoub B., Denoeud F., Liu S., Parkin I.A., Tang H., Wang X., Chiquet J., Belcram H., Tong C., Samans B. (2014). Early allopolyploid evolution in the post-Neolithic *Brassica napus* oilseed genome. Science.

[32] Parkin I.A.P., Koh C., Tang H., Robinson S.J., Kagale S., E Clarke W., Town C.D., Nixon J., Krishnakumar V., Bidwell S.L. (2014). Transcriptome and methylome profiling reveals relics of genome dominance in the mesopolyploid *Brassica oleracea*. Genome Biol..

[33] Stotz H.U., Harvey P.J., Haddadi P., Mashanova A., Kukol A., Larkan N.J., Borhan M.H., Fitt B.D.L., Raman H. (2018). Genomic evidence for genes encoding leucine-rich repeat receptors linked to resistance against the eukaryotic extra- and intracellular *Brassica napus* pathogens *Leptosphaeria maculans* and *Plasmodiophora brassicae*. PLoS ONE.

[34] Inturrisi F., Bayer P.E., Cantila A.Y., Tirnaz S., Edwards D., Batley J. (2022). In silico integration of disease resistance QTL, genes and markers with the *Brassica juncea* physical map. Mol. Breed..

[35] Lee J., Izzah N.K., Jayakodi M., Perumal S., Joh H.J., Lee H.J., Lee S.-C., Park J.Y., Yang K.-W., Nou I.-S. (2015). Genome-wide SNP identification and QTL mapping for black rot resistance in cabbage. BMC Plant Biol..

[36] Ferdous M.J., Hossain M.R., Park J.-I., Kim H.-T., Robin A.H.K., Natarajan S., Biswas M.K., Jung H.-J., Nou I.-S. (2020). In silico characterization and expression of disease-resistance-related genes within the collinear region of *Brassica napus* blackleg resistant locus *LepR1* in *B. oleracea*. J. Gen. Plant Pathol..

[37] Hossain M.R., Ferdous M.J., Park J.I., Robin A.H.K., Natarajan S., Jung H.-J., Kim H.-T., Nou I.-S. (2020). In-silico identification and differential expression of putative disease resistance-related genes within the collinear region of *Brassica napus* blackleg resistance locus *LepR2*′ in *Brassica oleracea*. Hortic. Environ. Biotechnol..

[38] Ferdous M.J., Hossain M.R., Park J.-I., Robin A.H.K., Natarajan S., Jesse D.M.I., Jung H.-J., Kim H.-T., Nou I.-S. (2020). In-silico identification and differential expressions of *LepR4*-syntenic disease resistance-related domain containing genes against blackleg causal fungus *Leptosphaeria maculans* in *Brassica oleracea*. Gene Rep..

[39] Ferdous M.J., Hossain M.R., Park J.-I., Robin A.H.K., Jesse D.M.I., Jung H.-J., Kim H.-T., Nou I.-S. (2019). Inheritance pattern and molecular markers for resistance to blackleg disease in cabbage. Plants.

[40] Chang A., Lamara M., Wei Y., Hu H., Parkin I.A.P., Gossen B.D., Peng G., Yu F. (2019). Clubroot resistance gene Rcr6 in *Brassica nigra* resides in a genomic region homologous to chromosome A08 in B. rapa. BMC Plant Biol..

[41] Yang H., Saad N.S.M., Ibrahim M.I., Bayer P.E., Neik T.X., Severn-Ellis A.A., Pradhan A., Tirnaz S., Edwards D., Batley J. (2021). Candidate Rlm6 resistance genes against *Leptosphaeria maculans* identified through a genome-wide association study in *Brassica juncea* (L.) Czern. Theor. Appl. Genet..

[42] Raman H., Raman R., Qiu Y., Zhang Y., Batley J., Liu S. (2021). The Rlm13 gene, a new player of *Brassica napus–Leptosphaeria maculans* interaction maps on chromosome C03 in canola. Front. Plant Sci..

[43] Letunic I., Bork P. (2021). Interactive Tree of Life (iTOL) v5: An online tool for phylogenetic tree display and annotation. Nucleic Acids Res..

[44] Bayer P.E., Golicz A.A., Tirnaz S., Chan C.K.K., Edwards D., Batley J. (2019). Variation in abundance of predicted resistance genes in the *Brassica oleracea* pangenome. Plant Biotechnol. J..

[45] Shiu S.H., Bleecker A.B. (2003). Expansion of the receptor-like kinase/Pelle gene family and receptor-like proteins in *Arabidopsis*. Plant Physiol..

[46] Meyers B.C., Kozik A., Griego A., Kuang H., Michelmore R.W. (2003). Genome-wide analysis of NBS-LRR-encoding genes in Arabidopsis. Plant Cell.

[47] Shiu S.H., Karlowski W.M., Pan R., Tzeng Y.H., Mayer K.F., Li W.H. (2004). Comparative analysis of the receptor-like kinase family in *Arabidopsis* and rice. Plant Cell.

[48] Zhou T., Wang Y., Chen J.Q., Araki H., Jing Z., Jiang K., Shen J., Tian D. (2004). Genome-wide identification of NBS genes in japonica rice reveals significant expansion of divergent non-TIR NBS-LRR genes. Mol. Genet. Genom..

[49] Fritz-Laylin L.K., Krishnamurthy N., Tör M., Sjölander K.V., Jones J.D. (2005). Phylogenomic analysis of the receptor-like proteins of rice and *Arabidopsis*. Plant Physiol..

[50] Li Y., Wei W., Feng J., Luo H., Pi M., Liu Z., Kang C. (2018). Genome re-annotation of the wild strawberry *Fragaria vesca* using extensive Illumina- and SMRT-based RNA-seq datasets. DNA Res..

[51] Yang H., Bayer P.E., Tirnaz S., Edwards D., Batley J. (2020). Genome-wide identification and evolution of receptor-like kinases (RLKs) and receptor-like proteins (RLPs) in *Brassica juncea*. Biology.

[52] Larkan N.J., Ma L., Haddadi P., Buchwaldt M., Parkin I.A.P., Djavaheri M., Borhan M.H. (2020). The *Brassica napus* wall-associated kinase-like (WAKL) gene *Rlm9* provides race-specific blackleg resistance. Plant J..

[53] McHale L., Tan X., Koehl P., Michelmore R.W. (2006). Plant NBS-LRR proteins: Adaptable guards. Genome Biol..

[54] Li X., Salman A., Guo C., Yu J., Cao S., Gao X., Li W., Li H., Guo Y. (2018). Identification and characterization of LRR-RLK family genes in potato reveal their involvement in peptide signaling of cell fate decisions and biotic/abiotic stress responses. Cells.

[55] Yuan N., Rai K.M., Balasubramanian V.K., Upadhyay S.K., Luo H., Mendu V. (2018). Genome-wide identification and characterization of LRR-RLKs reveal functional conservation of the SIF subfamily in cotton (*Gossypium hirsutum*). BMC Plant Biol..

[56] Shumayla Sharma S., Kumar R., Mendu V., Singh K., Upadhyay S.K. (2016). Genomic dissection and expression profiling revealed functional divergence in *Triticum aestivum* leucine rich repeat receptor like kinases (TaLRRKs). Front. Plant Sci..

[57] Sun X., Wang G.L. (2011). Genome-wide identification, characterization and phylogenetic analysis of the rice LRR-kinases. PLoS ONE.

[58] Tomé F., Nägele T., Adamo M., Garg A., Marco-Llorca C., Nukarinen E., Pedrotti L., Peviani A., Simeunovic A., Tatkiewicz A. (2014). The low energy signaling network. Front. Plant Sci..

[59] Narusaka M., Shirasu K., Noutoshi Y., Kubo Y., Shiraishi T., Iwabuchi M., Narusaka Y. (2009). *RRS1* and *RPS4* provide a dual resistance-gene system against fungal and bacterial pathogens. Plant J..

[60] Zhang B., Liu M., Wang Y., Yuan W., Zhang H. (2022). Plant NLRs: Evolving with pathogen effectors and engineerable to improve resistance. Front. Microbiol..

[61] Blommaert J. (2020). Genome size evolution: Towards new model systems for old questions. Proc. R. Soc. B Biol. Sci..

[62] Lu H., Giordano F., Ning Z. (2016). Oxford Nanopore MinION sequencing and genome assembly. Genom. Proteom. Bioinform..

[63] Mueller R.L., Jockusch E.L. (2018). Jumping genomic gigantism. Nat. Ecol. Evol..

[64] Shen C.-H. (2019). Chapter 5—The Genome. Diagnostic Molecular Biology.

[65] Gebhardt C., Valkonen J.P. (2001). Organization of genes controlling disease resistance in the potato genome. Annu. Rev. Phytopathol..

[66] Lozano R., Ponce O., Ramirez M., Mostajo N., Orjeda G. (2012). Genome-wide identification and mapping of NBS-encoding resistance genes in *Solanum tuberosum* group Phureja. PLoS ONE.

[67] Ameline-Torregrosa C., Wang B.B., O’Bleness M.S., Deshpande S., Zhu H., Roe B., Young N.D., Cannon S.B. (2008). Identification and characterization of nucleotide-binding site-leucine-rich repeat genes in the model plant *Medicago truncatula*. Plant Physiol..

[68] Yu J., Tehrim S., Zhang F., Tong C., Huang J., Cheng X., Dong C., Zhou Y., Qin R., Hua W. (2014). Genome-wide comparative analysis of NBS-encoding genes between *Brassica* species and *Arabidopsis thaliana*. BMC Genom..

[69] Golicz A.A., Bayer P.E., Barker G.C., Edger P.P., Kim H., Martinez P.A., Chan C.K.K., Severn-Ellis A., McCombie W.R., Parkin I.A. (2016). The pangenome of an agronomically important crop plant *Brassica oleracea*. Nat. Commun..

[70] Zhang Y.M., Shao Z.Q., Wang Q., Hang Y.Y., Xue J.Y., Wang B., Chen J.Q. (2016). Uncovering the dynamic evolution of nucleotide-binding site-leucine-rich repeat (NBS-LRR) genes in Brassicaceae. J. Integr. Plant Biol..

[71] Dolatabadian A., Bayer P.E., Tirnaz S., Hurgobin B., Edwards D., Batley J. (2020). Characterisation of disease resistance genes in the *Brassica napus* pangenome reveals significant structural variation. Plant Biotechnol. J..

[72] Alamery S., Tirnaz S., Bayer P., Tollenaere R., Chaloub B., Edwards D., Batley J. (2018). Genome-wide identification and comparative analysis of NBS-LRR resistance genes in *Brassica napus*. Crop Pasture Sci..

[73] Ma Y., Chhapekar S.S., Lu L., Oh S., Singh S., Kim C.S., Kim S., Choi G.J., Lim Y.P., Choi S.R. (2021). Genome-wide identification and characterization of NBS-encoding genes in *Raphanus sativus* L. and their roles related to *Fusarium oxysporum* resistance. BMC Plant Biol..

[74] Mizuno H., Katagiri S., Kanamori H., Mukai Y., Sasaki T., Matsumoto T., Wu J. (2020). Evolutionary dynamics and impacts of chromosome regions carrying R-gene clusters in rice. Sci. Rep..

[75] Perochon A., Benbow H.R., Ślęczka-Brady K., Malla K.B., Doohan F.M. (2021). Analysis of the chromosomal clustering of *Fusarium*-responsive wheat genes uncovers new players in the defense against head blight disease. Sci. Rep..

[76] van Wersch S., Li X. (2019). Stronger when together: Clustering of plant NLR disease resistance genes. Trends Plant Sci..

[77] Hulbert S.H., Webb C.A., Smith S.M., Sun Q. (2001). Resistance gene complexes: Evolution and utilization. Annu. Rev. Phytopathol..

[78] De Araújo A.C., Fonseca F.C.D.A., Cotta M.G., Alves G.S.C., Miller R.N.G. (2019). Plant NLR receptor proteins and their potential in the development of durable genetic resistance to biotic stresses. Biotechnol. Res. Innov..

[79] Sanseverino W., Hermoso A., D’aLessandro R., Vlasova A., Andolfo G., Frusciante L., Lowy E., Roma G., Ercolano M.R. (2013). PRGdb 2.0: Towards a community-based database model for the analysis of R-genes in plants. Nucleic Acids Res..

[80] Caldwell K.S., Michelmore R.W. (2009). Arabidopsis thaliana genes encoding defense signaling and recognition proteins exhibit contrasting evolutionary dynamics. Genetics.

[81] Song X., Wei Y., Xiao D., Gong K., Sun P., Ren Y., Yuan J., Wu T., Yang Q., Li X. (2021). *Brassica carinata* genome characterization clarifies U’s triangle model of evolution and polyploidy in *Brassica*. Plant Physiol..

[82] Zheng M.S., Takahashi H., Miyazaki A., Hamamoto H., Yamaguchi I., Kusano T., Shah J. (2004). Up-regulation of *Arabidopsis thaliana NHL10* in the hypersensitive response to cucumber mosaic virus infection and in senescing leaves is controlled by signalling pathways that differ in salicylate involvement. Planta.

[83] Tang D., Wang G., Zhou J.M. (2017). Receptor kinases in plant-pathogen interactions: More than pattern recognition. Plant Cell.

[84] Dodds P.N., Chen J., Outram M.A. (2024). Pathogen perception and signaling in plant immunity. Plant Cell.

[85] Kibby E.M., Conte A.N., Burroughs A.M., Nagy T.A., Vargas J.A., Whalen L.A., Aravind L., Whiteley A.T. (2023). Bacterial NLR-related proteins protect against phage. Cell.

[86] Duxbury Z., Wu C.-H., Ding P. (2021). A comparative overview of the intracellular guardians of plants and animals: NLRs in innate immunity and beyond. Annu. Rev. Plant Biol..

[87] Kourelis J., Sakai T., Adachi H., Kamoun S. (2021). RefPlantNLR is a comprehensive collection of experimentally validated plant disease resistance proteins from the NLR family. PLoS Biol..

[88] Meyers B., Dickerman A., Michelmore R., Sivaramakrishnan S., Sobral B., Young N. (1999). Plant disease resistance genes encode members of an ancient and diverse protein family within the nucleotide-binding superfamily. Plant J..

[89] Larkan N.J., Lydiate D.J., Parkin I.A.P., Nelson M.N., Epp D.J., Cowling W.A., Rimmer S.R., Borhan M.H. (2013). The *Brassica napus* blackleg resistance gene *LepR3* encodes a receptor-like protein triggered by the *Leptosphaeria maculans* effector *AVRLM1*. New Phytol..

[90] Larkan N.J., Ma L., Borhan M.H. (2015). The *Brassica napus* receptor-like protein *RLM2* is encoded by a second allele of the *LepR3/Rlm2* blackleg resistance locus. Plant Biotechnol. J..

[91] Ma L., Borhan M.H. (2015). The receptor-like kinase *SOBIR1* interacts with *Brassica napus LepR3* and is required for *Leptosphaeria maculans AvrLm1*-triggered immunity. Front. Plant Sci..

[92] Boutrot F., Zipfel C. (2017). Function, discovery, and exploitation of plant pattern recognition receptors for broad-spectrum disease resistance. Annu. Rev. Phytopathol..

[93] Wang Y., VandenLangenberg K., Wehner T.C., Weng Y. (2014). QTLs for downy mildew resistance and their association with LRR-RLK/RLP resistance gene homologs in cucumber. Cucurbitaceae.

