# Supplementary material for: Exploring Cloned Disease Resistance Gene Homologues and Resistance Gene Analogues in Brassica nigra, Sinapis arvensis, and Sinapis alba: Identification, Characterisation, Distribution, and Evolution
Source: Genes (Basel). 2025 Jul 22;16(8):849. doi: 10.3390/genes16080849 (PMC12385795; doi:10.3390/genes16080849)
Supplement: Supplementary file 1 [file genes-16-00849-s001.zip › Supplementary figures new.pdf]

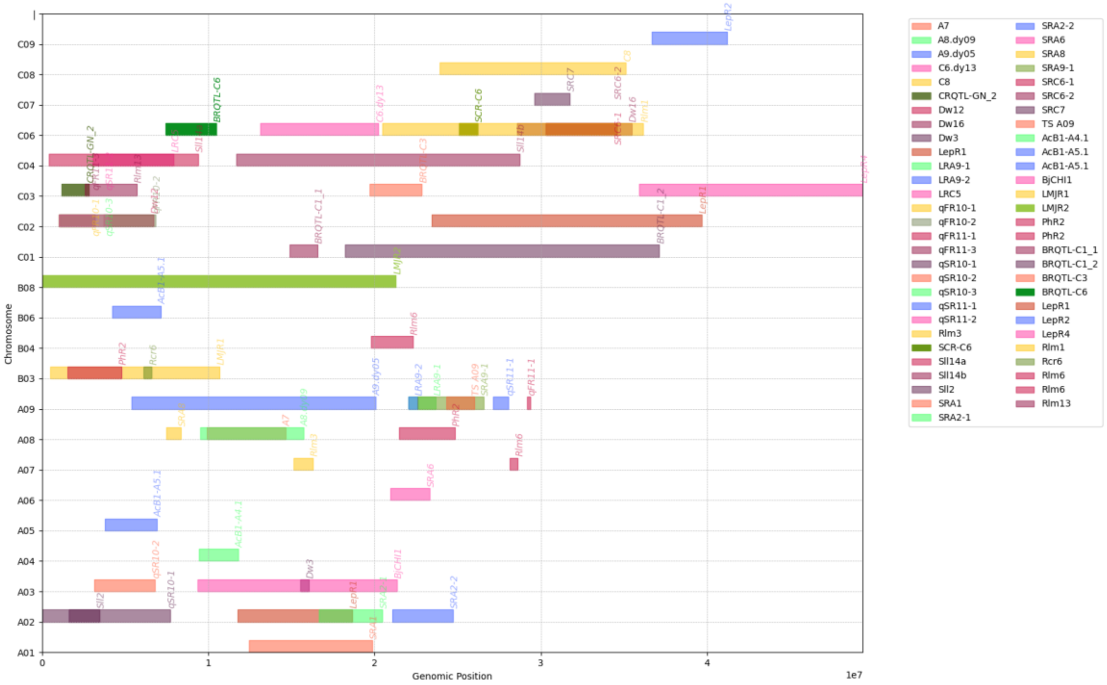

Supplementary figure S1: Distribution and overlap of 57 QTL associated with disease resistance in *Brassica* crops. QTL are mapped across 21 chromosomes and confer resistance to blackleg (BL), black rot (BR), clubroot (CR), hypocotyl rot (HR), Sclerotinia stem rot (SSR), and white rust (WR) (see Table 1 for details). Overlapping regions among these QTL are illustrated.

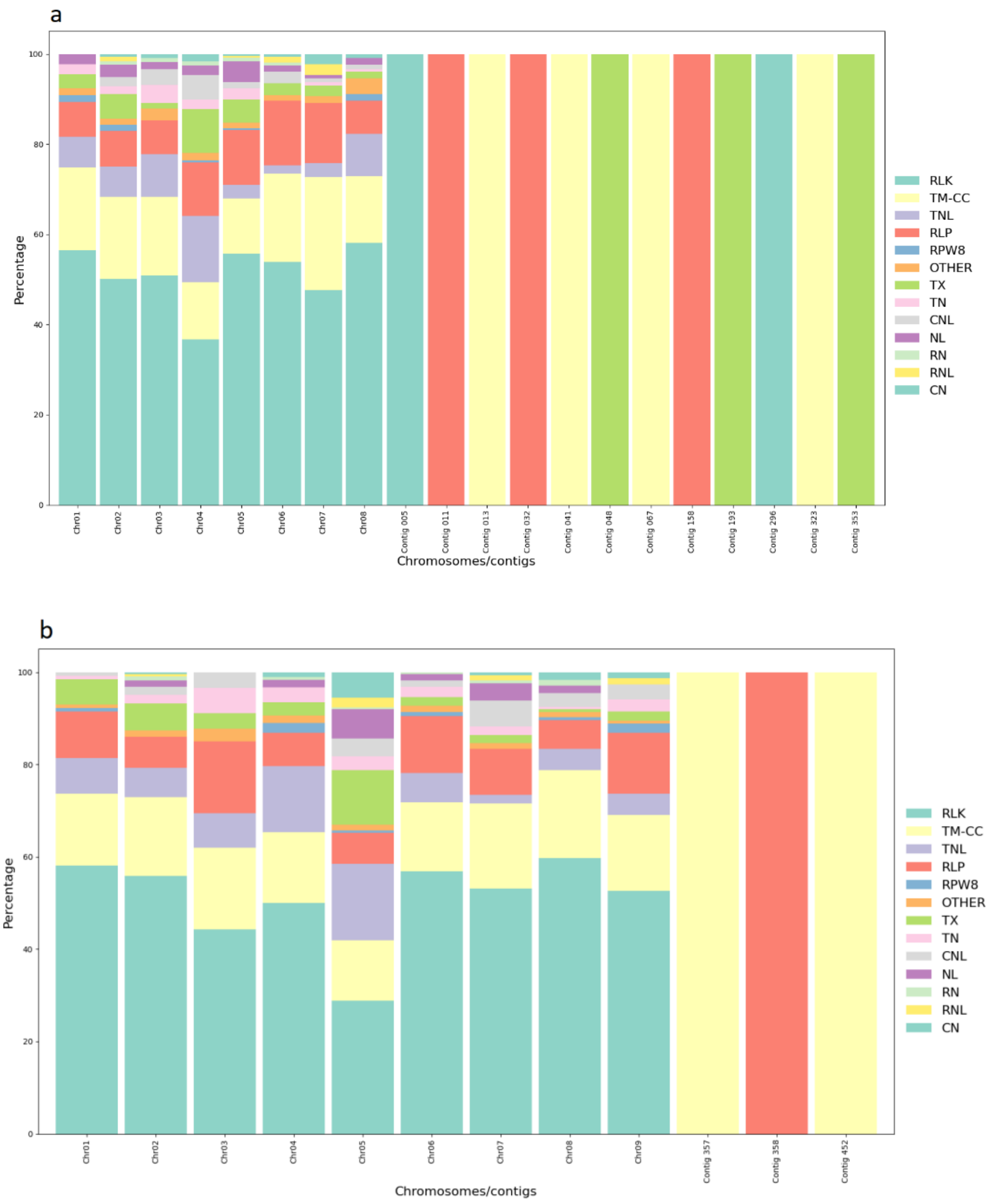
a
Percentage
Chromosomes/contigs
Chr01
Chr02
Chr03
Chr04
Chr05
Chr06
Chr07
Chr08
Contig 005
Contig 011
Contig 013
Contig 032
Contig 041
Contig 048
Contig 067
Contig 158
Contig 193
Contig 296
Contig 323
Contig 353
RLK
TM-CC
TNL
RLP
RPW8
OTHER
TX
TN
CNL
NL
RN
RNL
CN
b
Percentage
Chromosomes/contigs
Chr01
Chr02
Chr03
Chr04
Chr05
Chr06
Chr07
Chr08
Chr09
Contig 357
Contig 358
Contig 452
RLK
TM-CC
TNL
RLP
RPW8
OTHER
TX
TN
CNL
NL
RN
RNL
CN

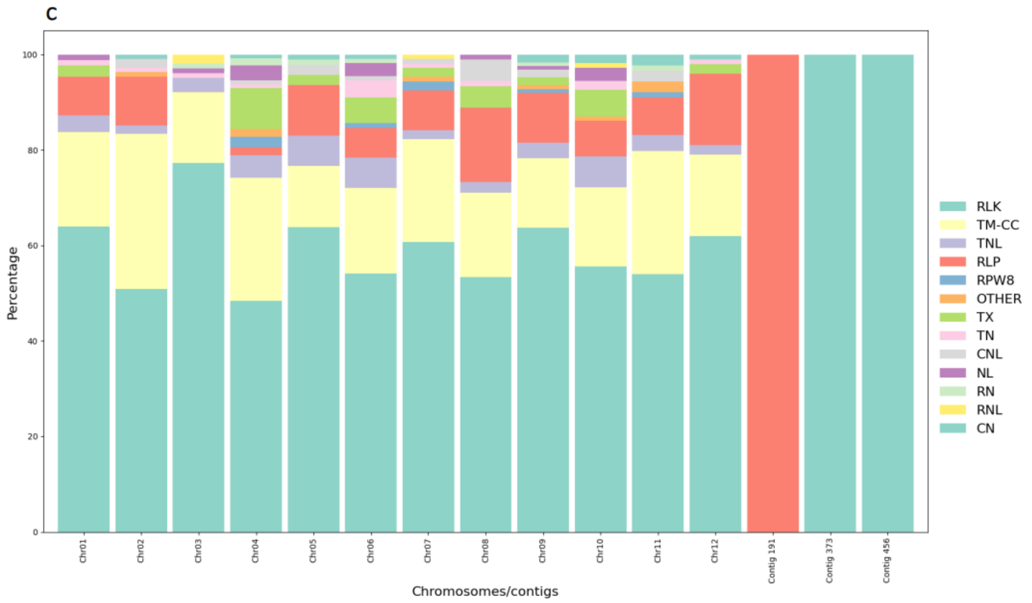

Supplementary figure S2: The percentage of RGAs on each chromosome and contigs. **a**: *B. nigra*, **b**: *S. arvensis* and **c**: *S. alba*. The coloured bars show the percentage of RGAs in chromosomes and contigs.

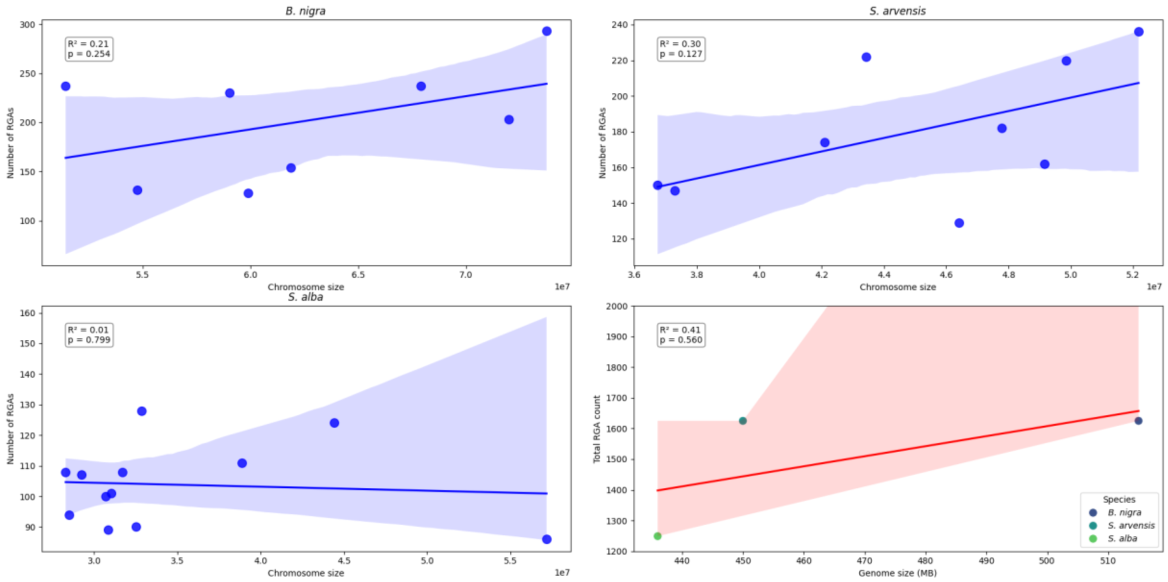

Supplementary figure S3: Relationship between chromosome and genome size with the number of RGAs.

a: *B. nigra*

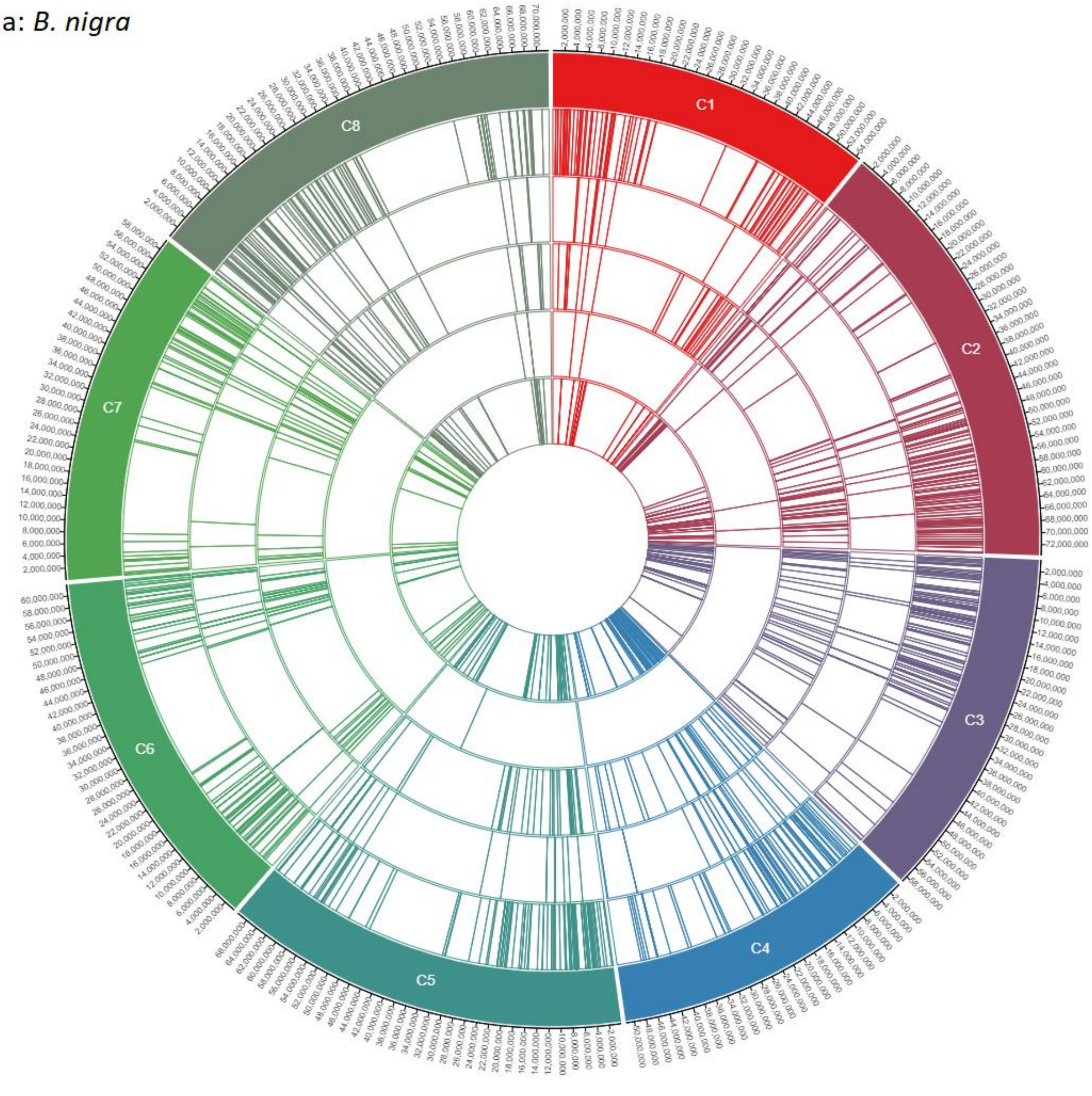

b: *S. arvensis*

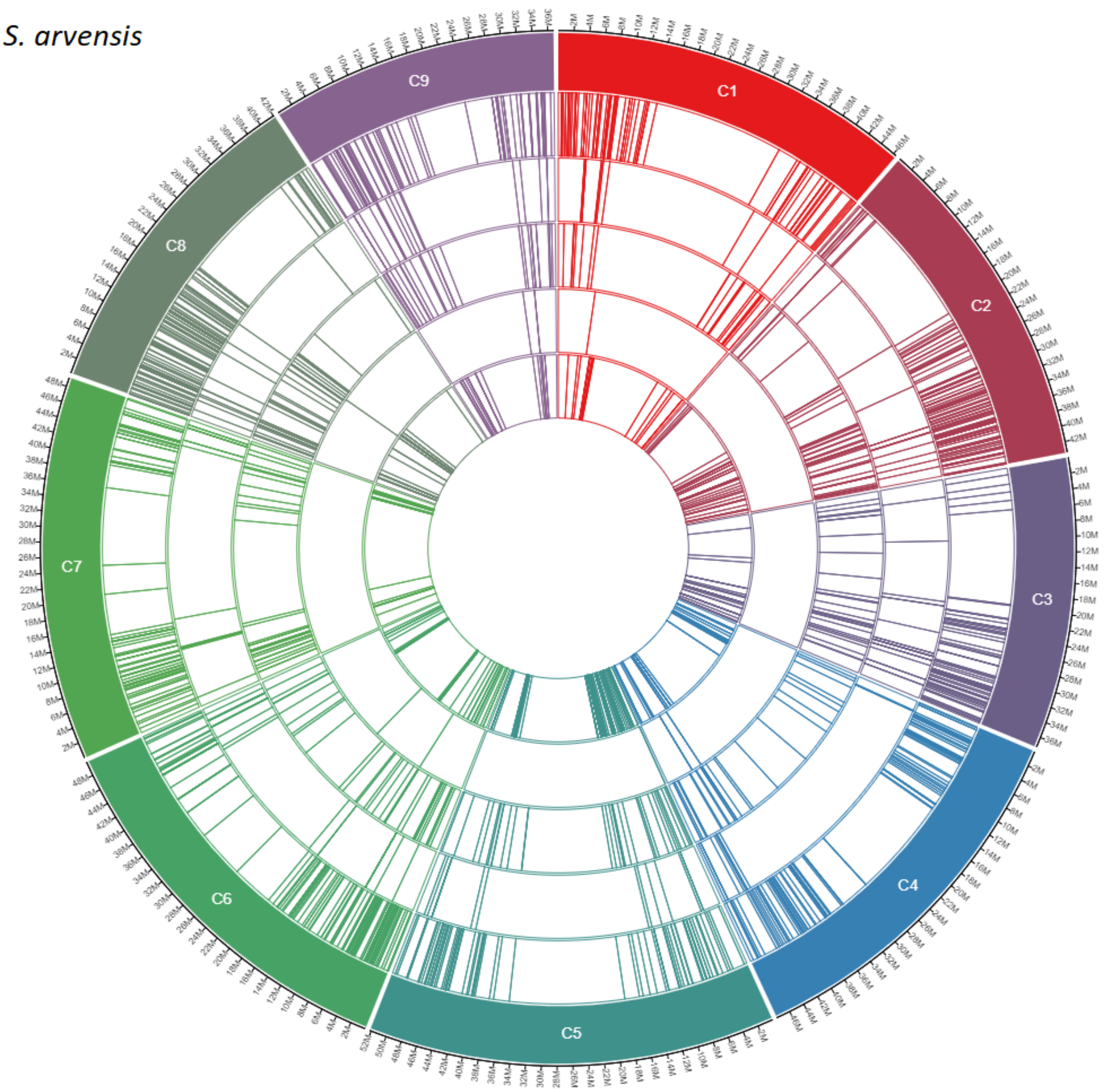

c: *S. alba*

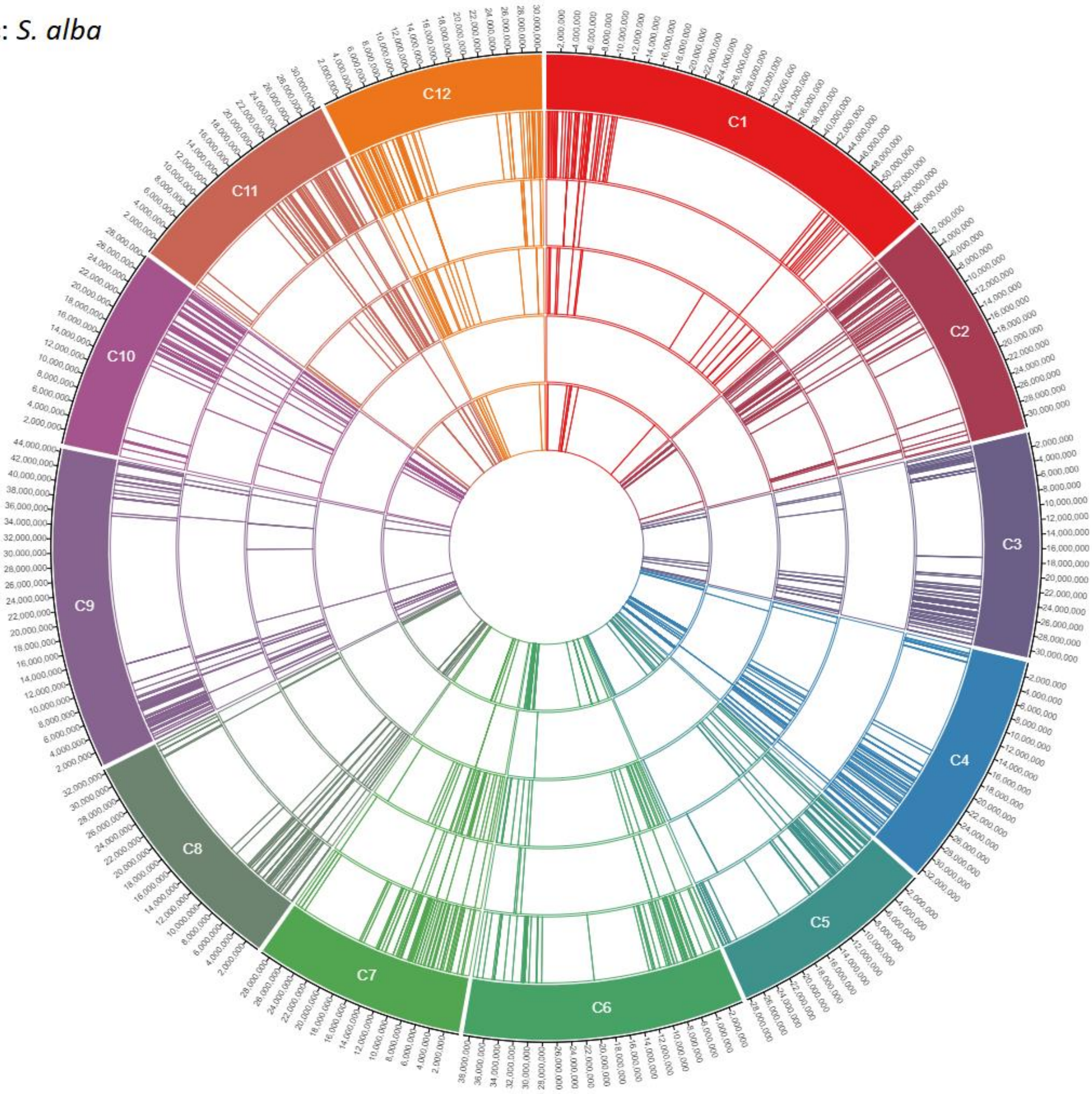

d: *B. nigra*

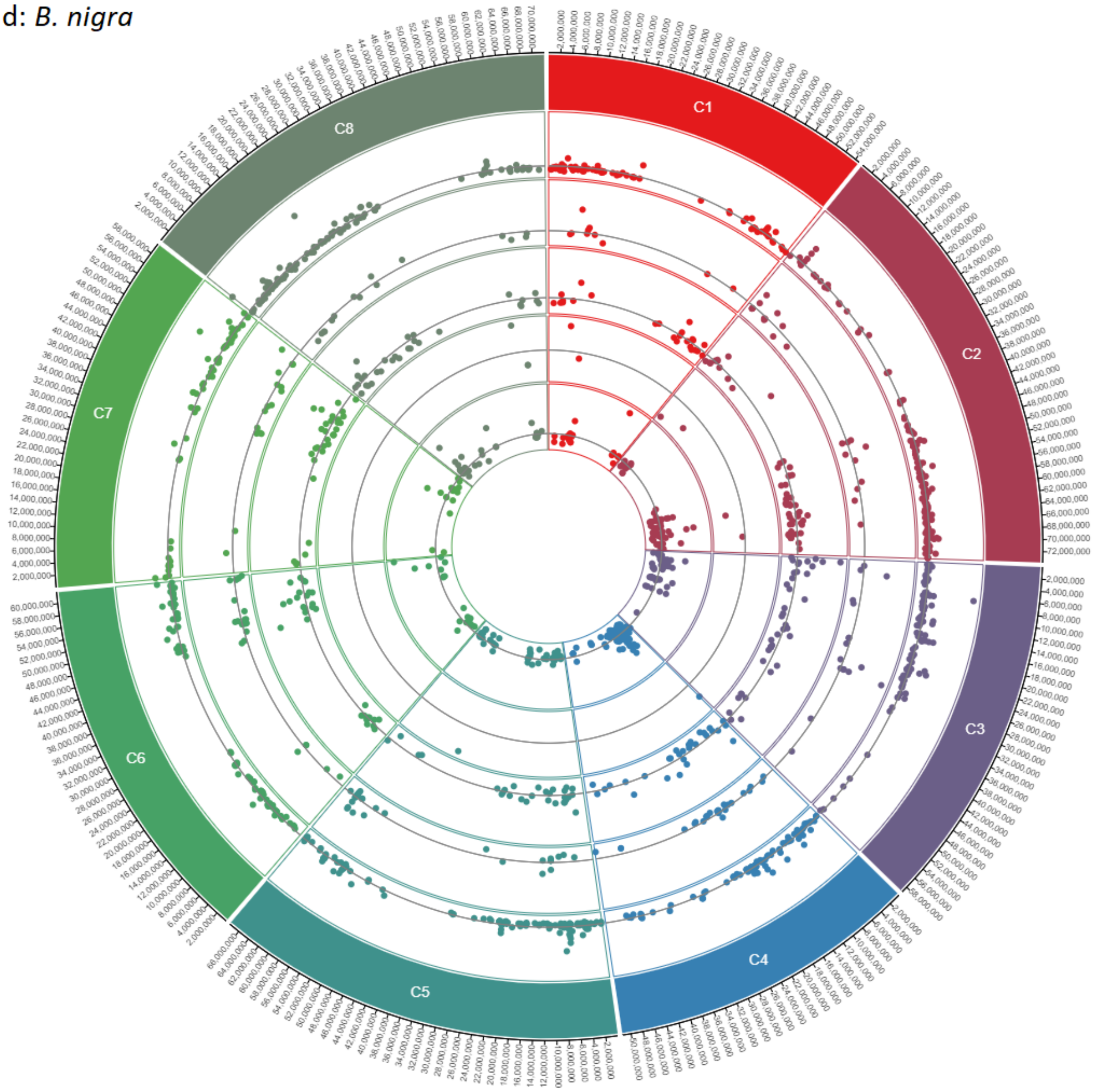

e: *S. arvensis*

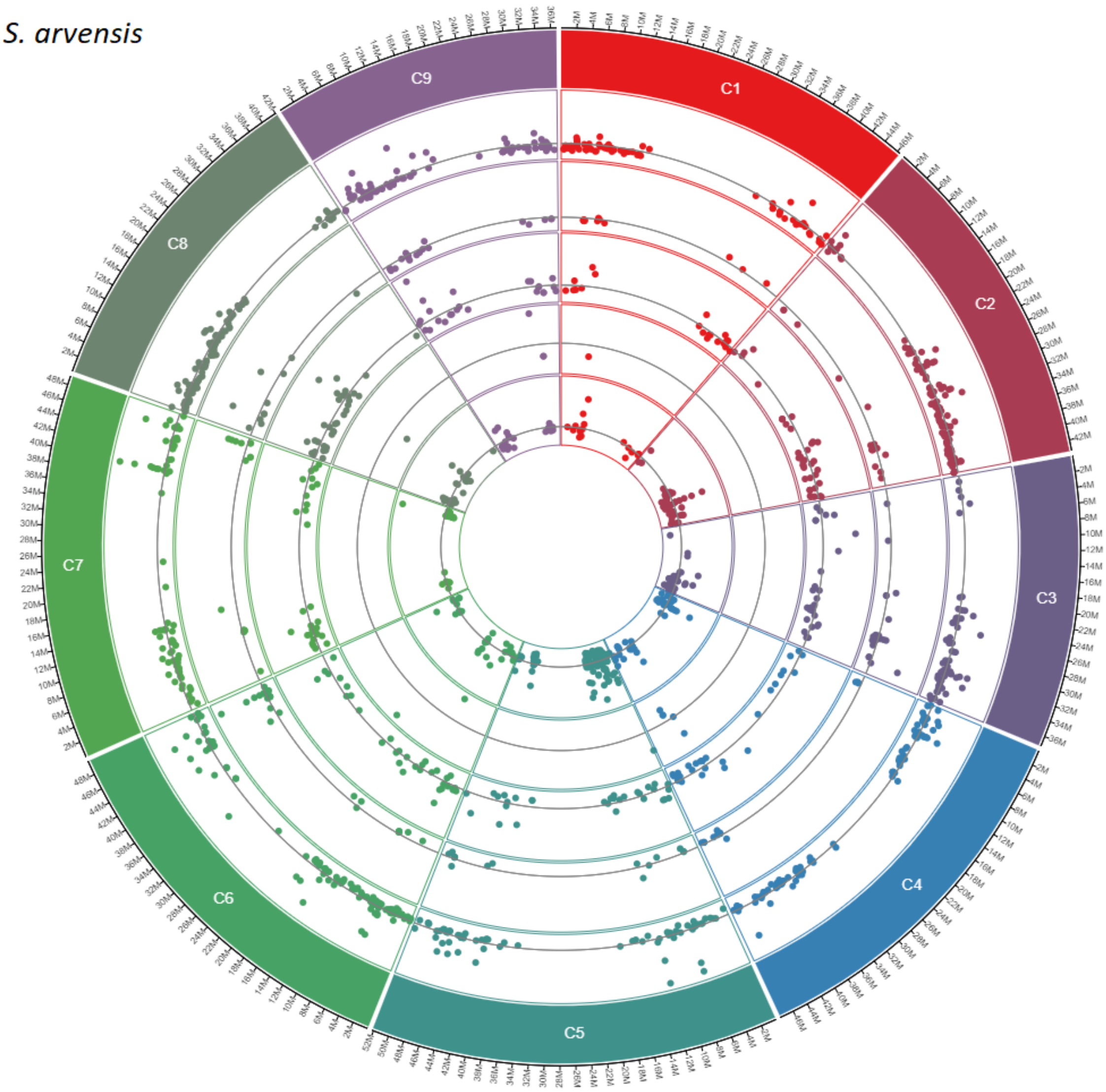

f: *S. alba*

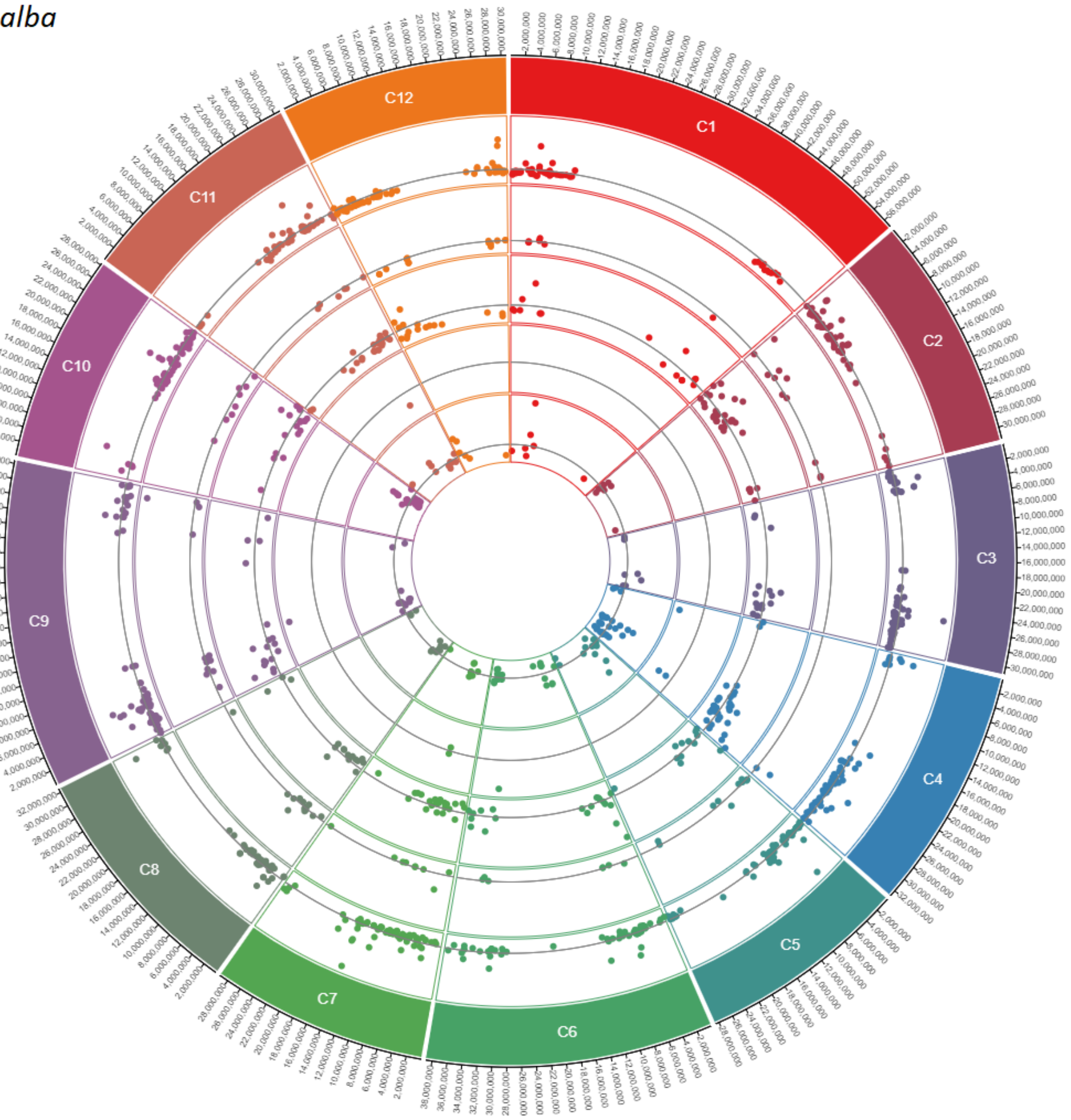

Supplementary figure S4: **a**, **b** and **c**: RGAs positions on the chromosomes. Outer to inner, RLK, RLP, TM-CC, RPW8 and NLR. **d, e** and **f:** RGAs size. Grey lines show average size.

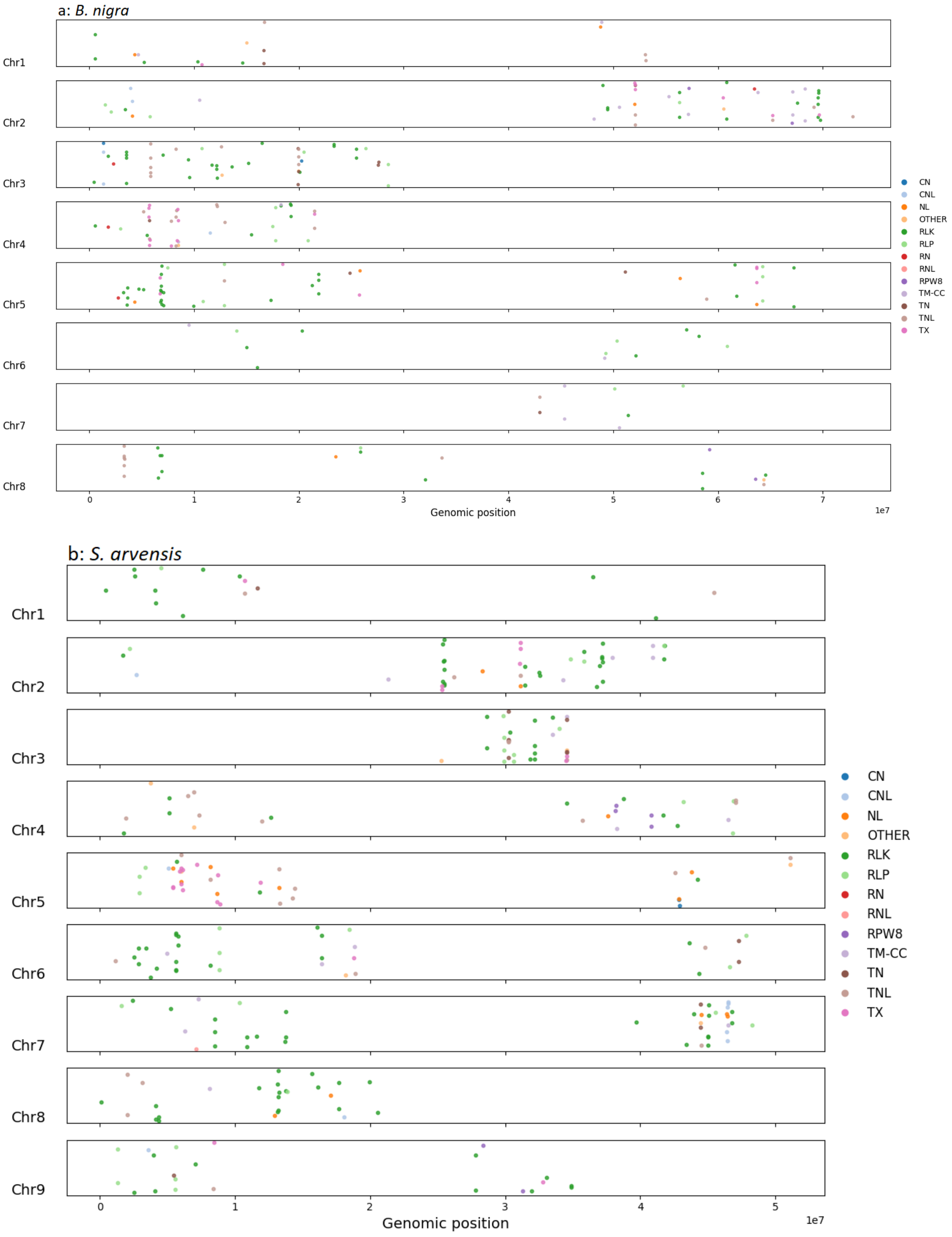
a: *B. nigra*
Chr1
Chr2
Chr3
Chr4
Chr5
Chr6
Chr7
Chr8
0
1
2
3
4
5
6
7
Genomic position
1e7
CN
CNL
NL
OTHER
RLK
RLP
RN
RNL
RPW8
TM-CC
TN
TNL
TX
b: *S. arvensis*
Chr1
Chr2
Chr3
Chr4
Chr5
Chr6
Chr7
Chr8
Chr9
0
1
2
3
4
5
Genomic position
1e7
CN
CNL
NL
OTHER
RLK
RLP
RN
RNL
RPW8
TM-CC
TN
TNL
TX

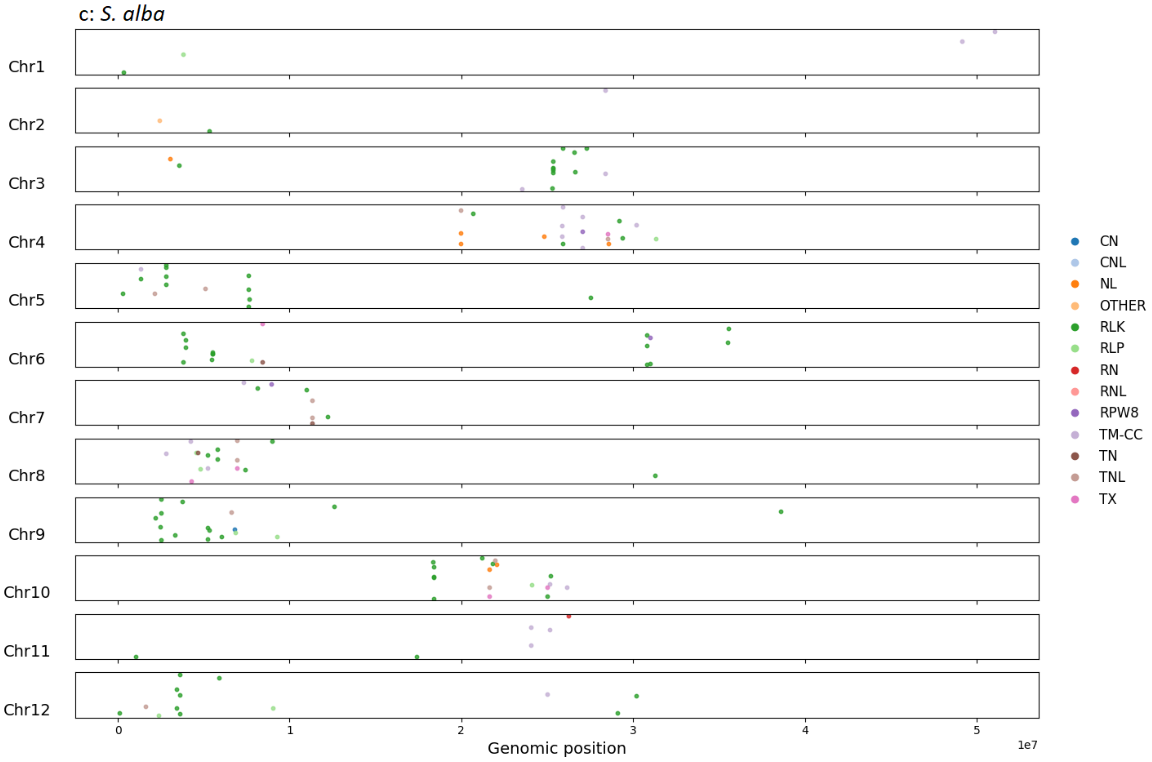

Supplementary figure S5: Spatial distribution of clustered RGAs across the *B. nigra* (**a**), *S. arvensis* (**b**) and *S. alba* (**c**) chromosomes. Clusters were defined as groups with a threshold distance of 10kb.

a: *B. nigra*

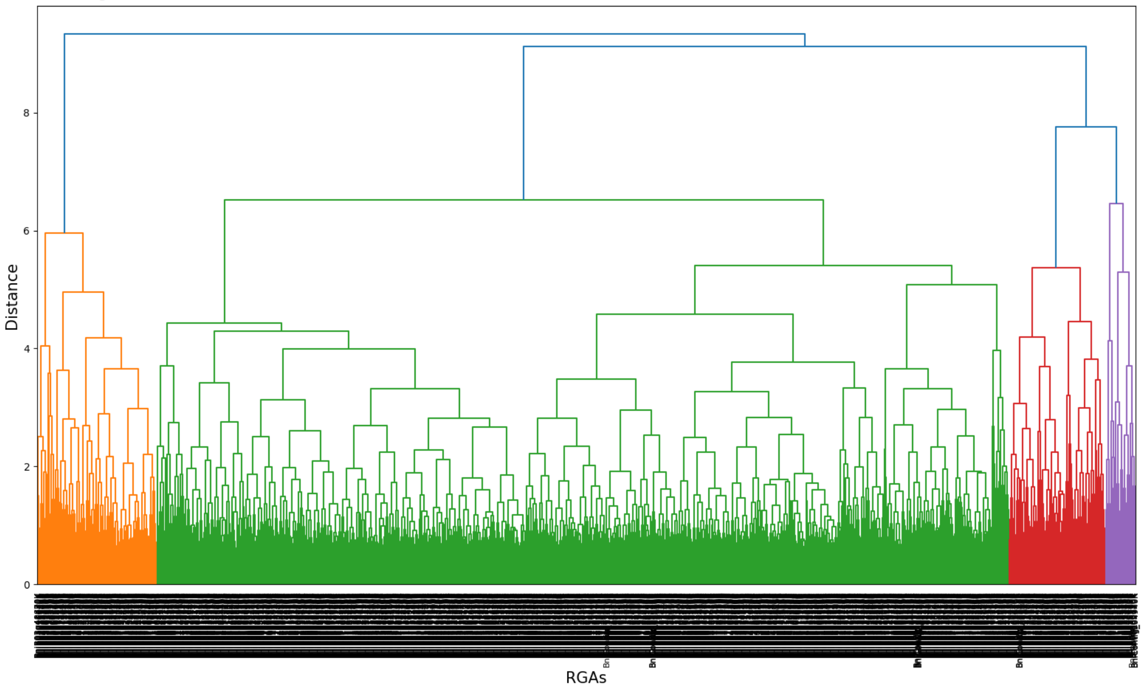

b: *S. arvensis*

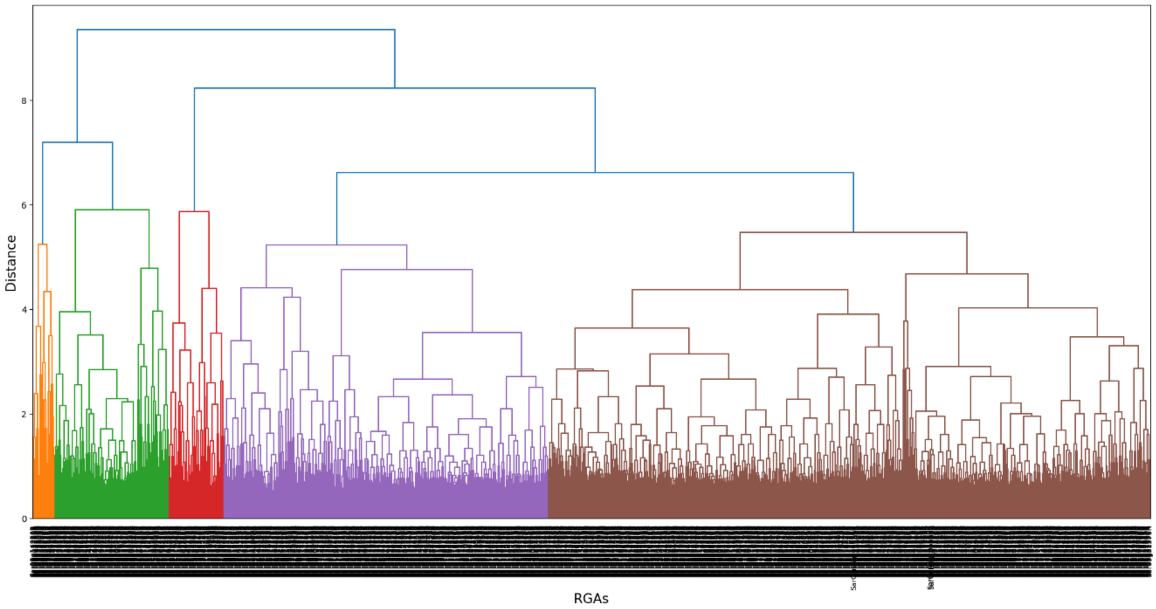

c: *S. alba*

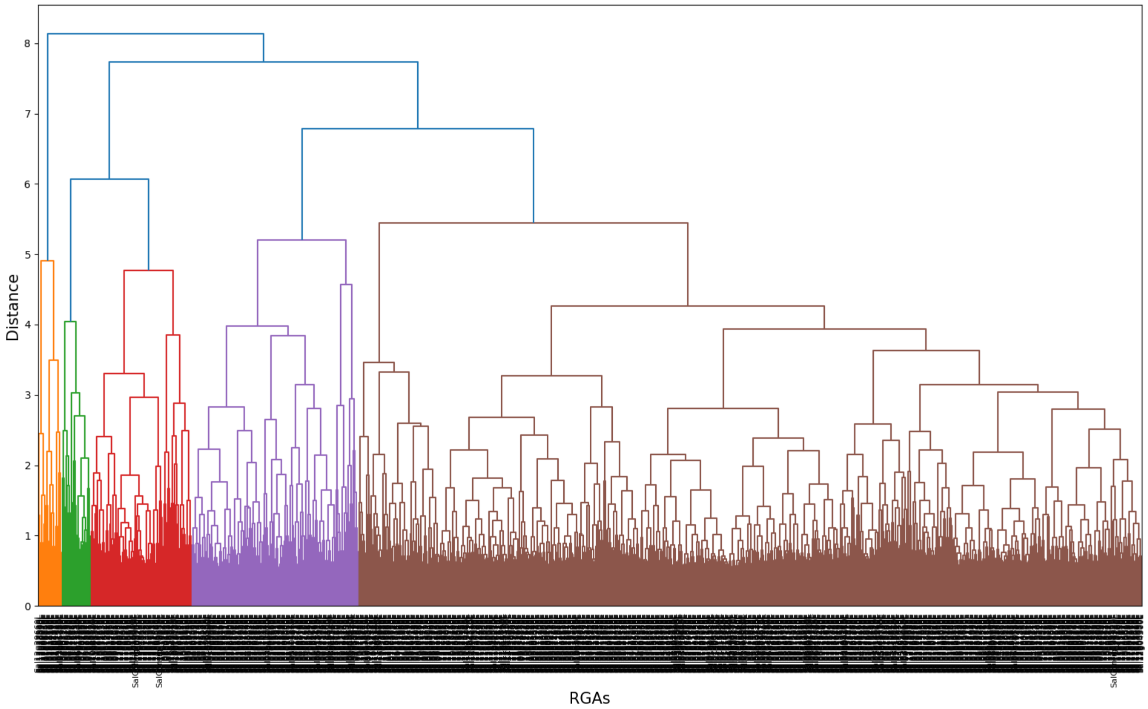

Supplementary figure S6: The hierarchical clustering dendrogram visually represents the relationships among the RGA sequences. In this dendrogram, genes are clustered based on their sequence similarities, with closer branches indicating higher similarity. The colour-coded branches emphasise the division of the RGAs into four (*B. nigra*) and five (*S. arvensis* and *S. alba*) discernible clusters.

a: *B. nigra* RLKs

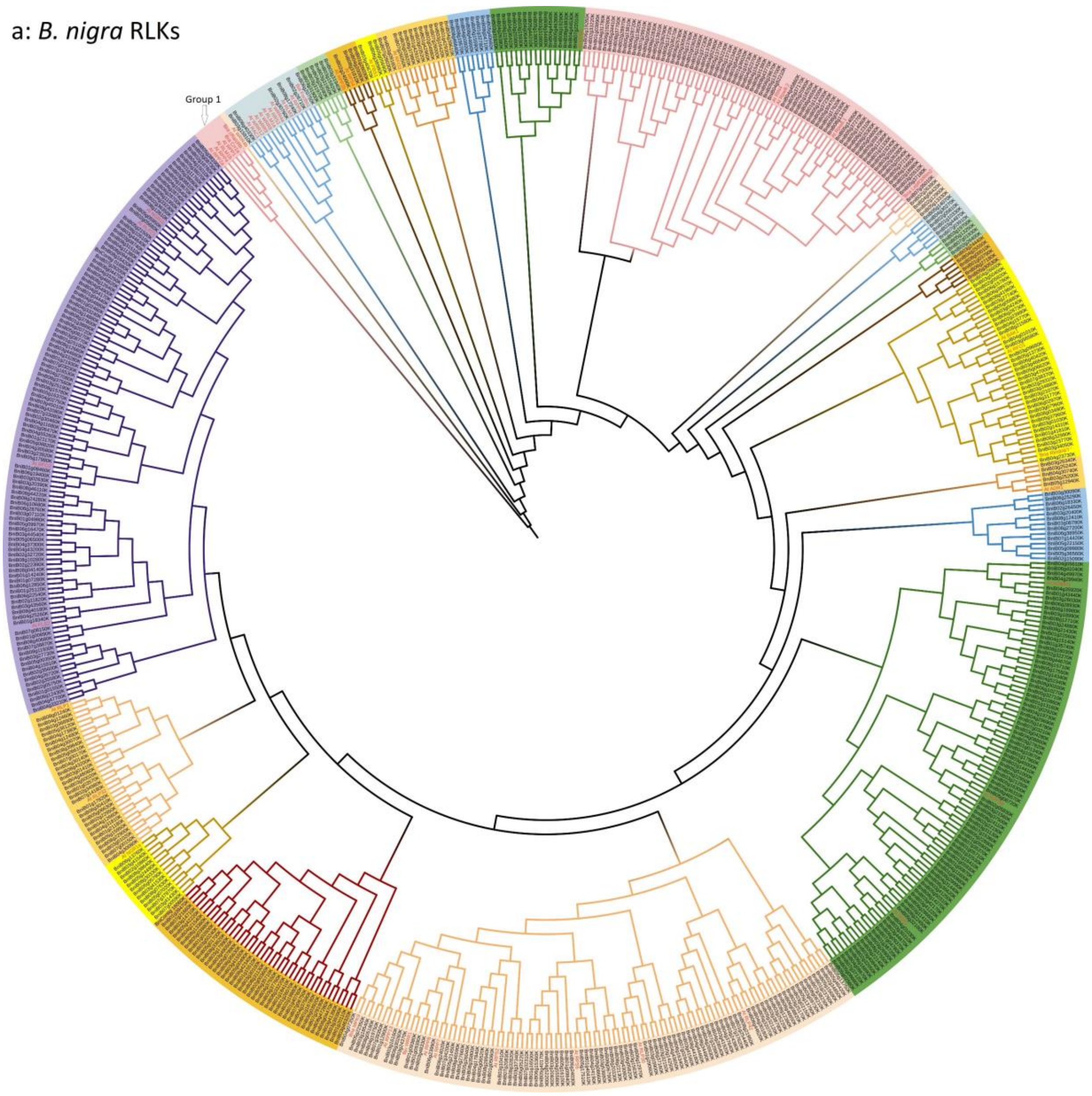

b: *B. nigra* RLPs

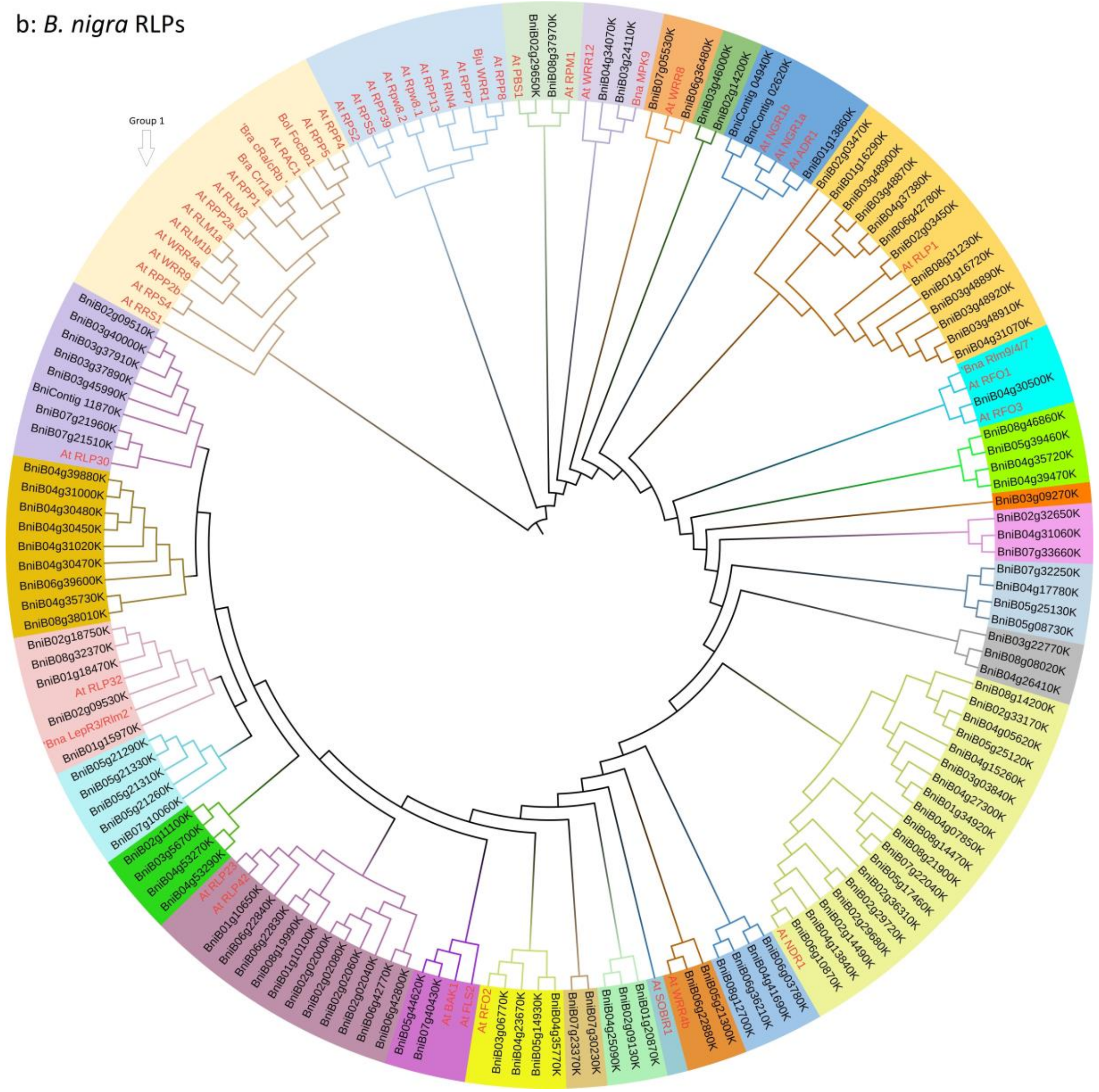

c: *S. arvensis* RLKs

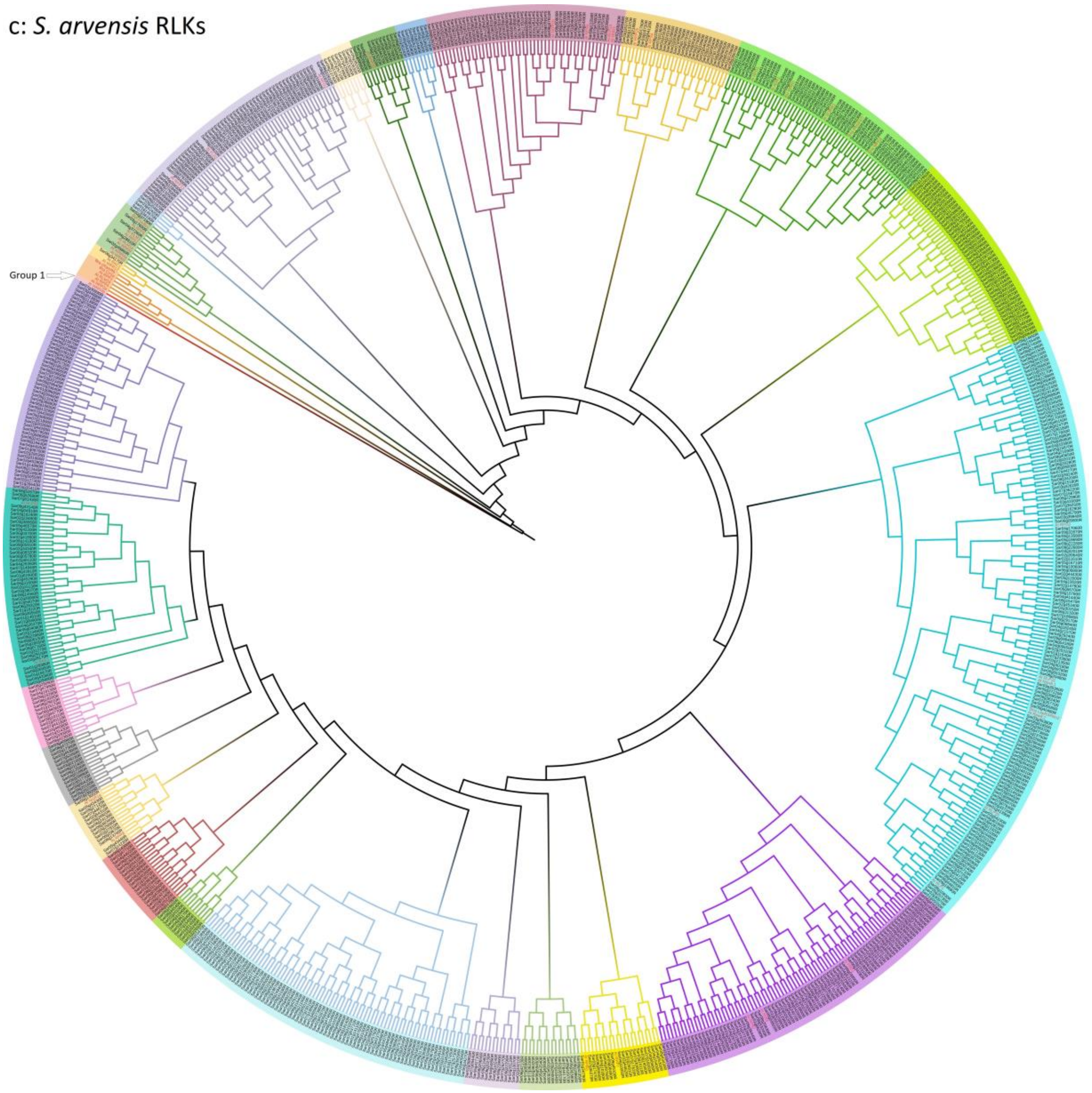

d: *S. arvensis* RLPs

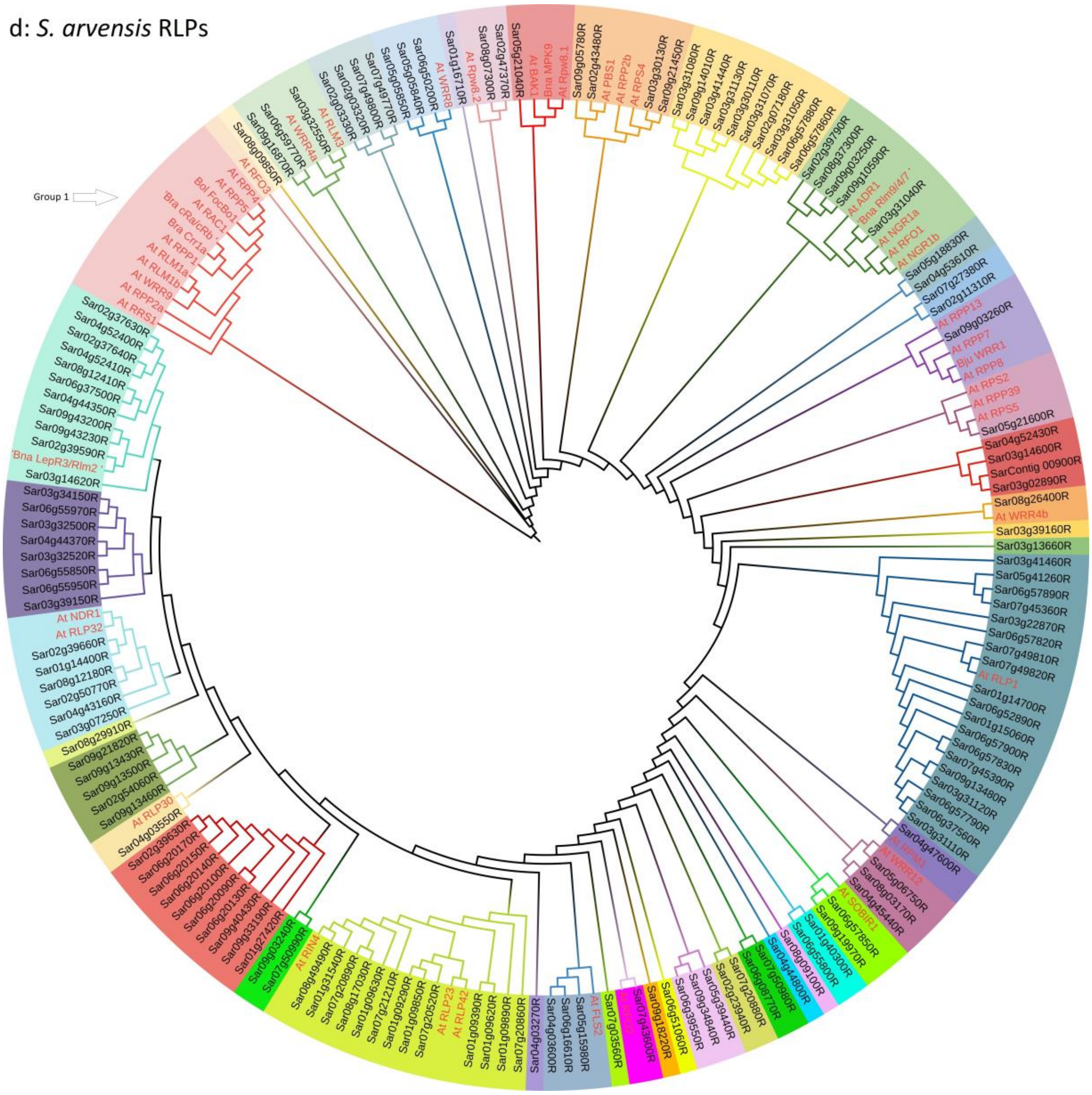

e: *S. alba* RLKs

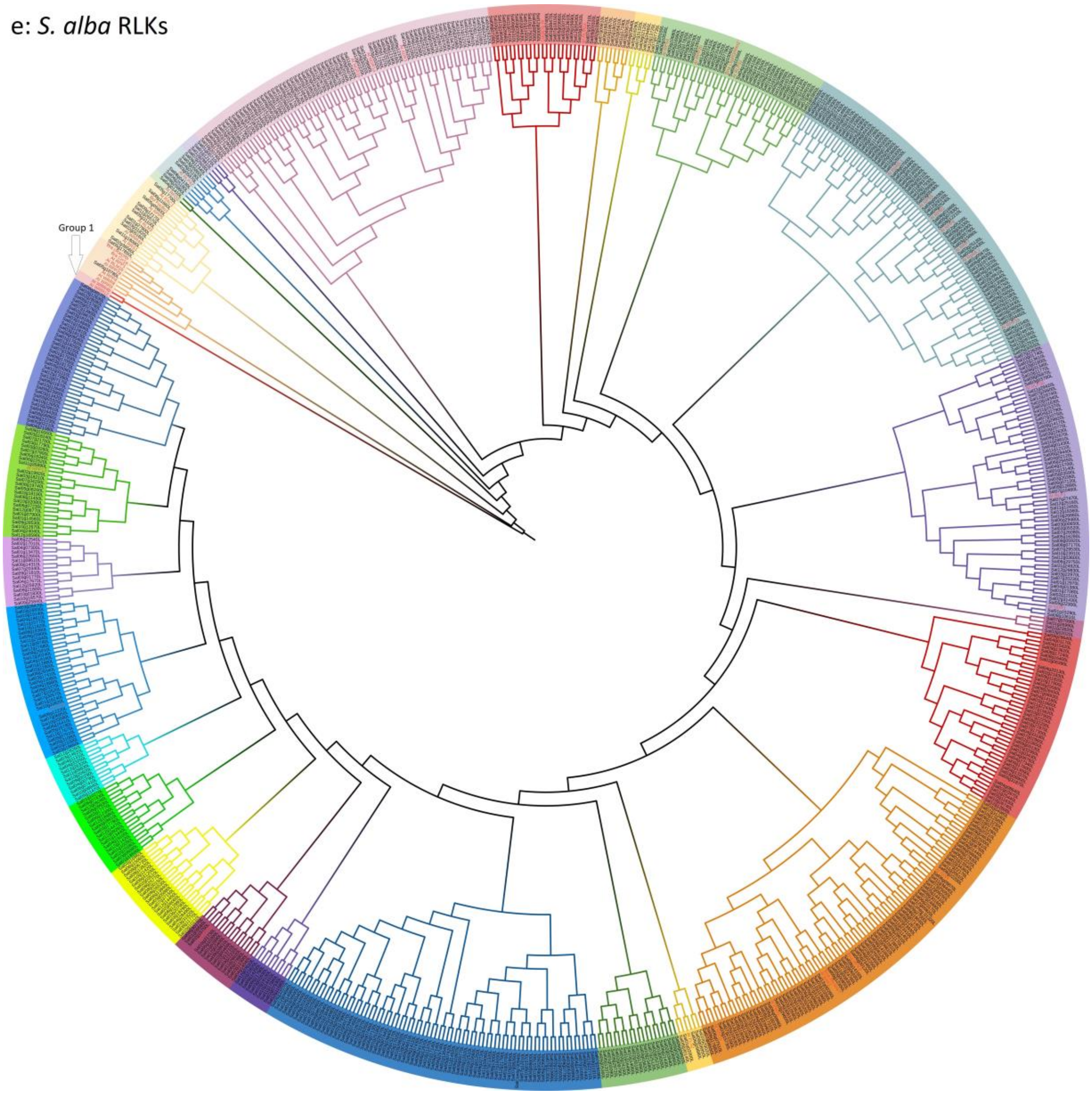

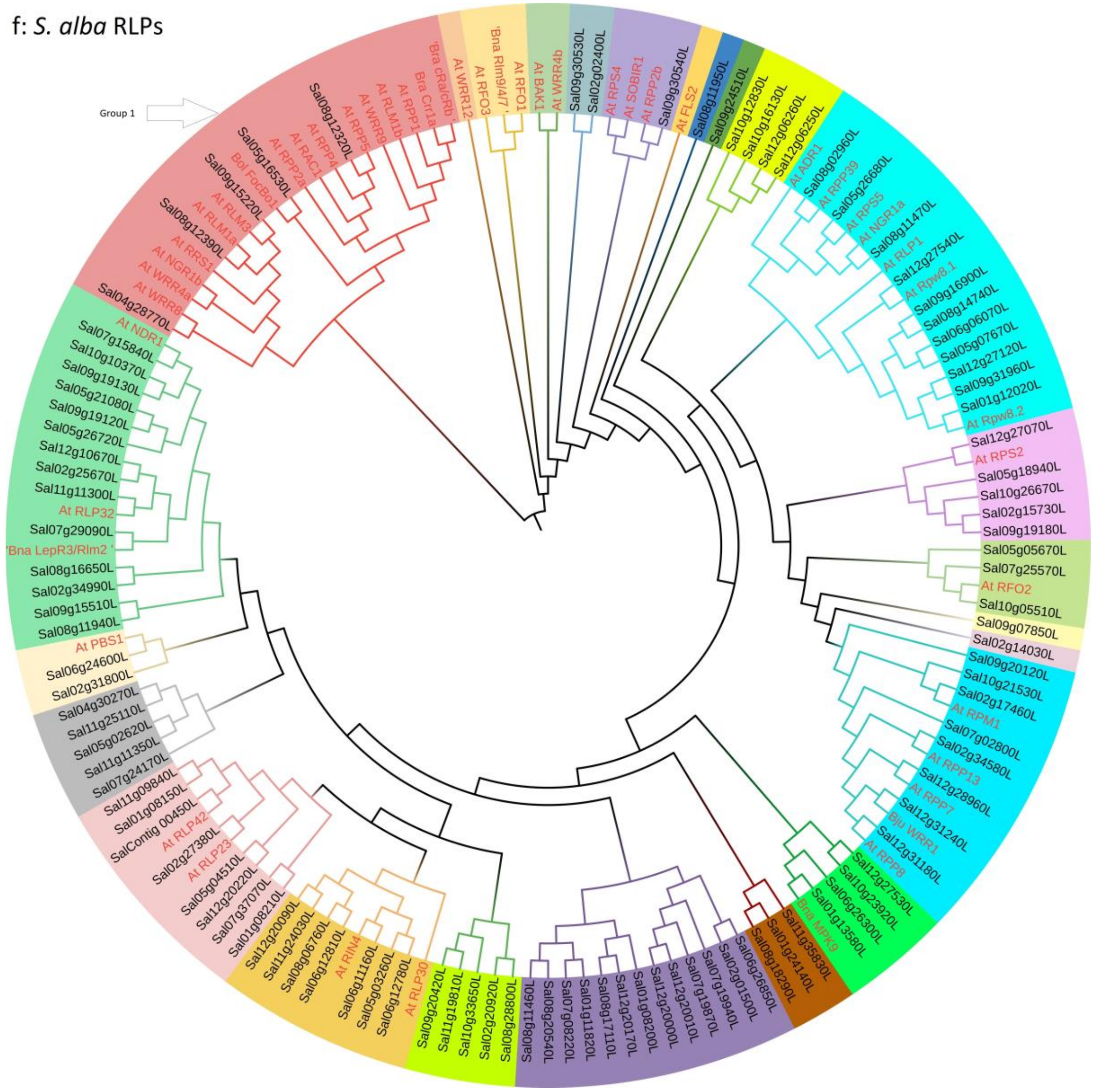

Supplementary figure S7: Phylogenetic analysis of 49 cloned RGAs, RLKs, and RLPs found within QTLs associated with disease resistance in *Brassica* crops. **a**, **b** and **c**: RLKs and cloned RGAs in *B. nigra*, *S. arvensis* and *S. alba*, respectively. **d, e** and **f:** RLPs and cloned RGAs in *B. nigra*, *S. arvensis* and *S. alba*, respectively. Group 1 is indicated by an arrow to distinguish it from the other groups, which are arranged in a clockwise direction.
